# Improving Interactions Between Health Technology Assessment Bodies and Regulatory Agencies: A Systematic Review and Cross-Sectional Survey on Processes, Progress, Outcomes, and Challenges

**DOI:** 10.3389/fmed.2020.582634

**Published:** 2020-10-16

**Authors:** Richard Ofori-Asenso, Christine E. Hallgreen, Marie Louise De Bruin

**Affiliations:** ^1^Copenhagen Centre for Regulatory Science (CORS), Department of Pharmacy, Faculty of Health and Medical Sciences, University of Copenhagen, Copenhagen, Denmark; ^2^Department of Epidemiology and Preventive Medicine, Monash University, Melbourne, VIC, Australia; ^3^Division of Pharmacoepidemiology & Clinical Pharmacology, Utrecht Institute for Pharmaceutical Sciences, Utrecht University, Utrecht, Netherlands

**Keywords:** HTA, regulatory approval, synergy, harmonization, collaboration synergy between HTA and regulatory agencies

## Abstract

The need to optimize drug development and facilitate faster access for patients has ignited discussions around the importance of improving interactions between health technology assessment (HTA) bodies and regulatory agencies. In this study, we conducted a systematic review to examine processes, progress, outcomes, and challenges of harmonization/interaction initiatives between HTA bodies and regulatory agencies. MEDLINE, EMBASE, and the International Pharmaceutical Abstracts database were searched up to 21 October 2019. Searches for gray literature (working papers, commissioned reports, policy documents, etc.) were performed via Google scholar and several institutional websites. An online cross-sectional survey was also conducted among HTA (*n* = 22) and regulatory agencies (*n* = 6) across Europe to supplement the systematic review. Overall, we found that while there are areas of divergence, there has been progress over time in narrowing the gap in evidentiary requirements for HTA bodies and regulatory agencies. Most regulatory agencies (4/6; 67%) and half (11/22, 50%) of the HTA bodies reported having a formal link for “collaborating” with the other. Several mechanisms such as early tripartite dialogues, parallel submissions (reviews), adaptive licensing pathways, and postauthorization data generation have been explored as avenues for improving collaboration. A number of pilot initiatives have shown positive effects of these models to reduce the time between regulatory and HTA decisions, which may translate into faster access for patients to life-saving therapies. Thus, future approaches aimed at improving harmonization/interaction between HTA bodies and regulatory agencies should build on these existing models/mechanisms while examining their long-term impacts. Several barriers including legal, organizational, and resource-related factors were also identified, and these need to be addressed to achieve greater alignment in the current regulatory and reimbursement landscape.

## Introduction

The transition of a product from benchside to clinical use involves several stages and engagements with different stakeholders (1). The first interaction is often with regulators who provide marketing authorization following satisfactory review of the product's risk–benefit profile (i.e., evaluation of safety, efficacy, and quality). Here, emphasis is usually placed on evidence generated from well-controlled studies (those with high internal validity), such as randomized controlled trials (RCTs) (2). Moreover, relative efficacy against an active comparator is often not a requirement, and placebo comparators are considered to provide simpler statistical and clinical interpretation (3, 4).

Once a product has gained marketing authorization, market access is further dictated by the particular healthcare system's financing mechanisms (5). Usually, payers relying on the assistance of health technology assessment (HTA) bodies decide whether to reimburse a product based on its relative value under current clinical practice scenarios (6). The value assessments usually focus on relative performance (such as relative safety, relative effectiveness, and cost effectiveness) of a technology against currently available clinical options. Unlike regulators who may accept short-term or surrogate outcomes, payers usually prefer long-term clinical outcomes (3, 7). Moreover, their assessment may involve a broader perspective such as the consideration of the potential social, legal, ethical, and political impacts of adopting the new technology (8, 9).

Given the differences in decision mandates of HTA/reimbursement bodies and regulatory agencies, their activities have been distinct from each other (Table 1). However, there are growing interests in aligning the activities of these institutions (12, 13). The interests in improving harmonization/alignment stem from criticisms that the current “silo-based model” is ill-equipped to drive innovation, that it hinders the rapid adoption of evolving clinical evidence, and that it delays timely patient access to life-saving technologies (3, 13). As an example, among all new medicines approved by the European Medicines Agency (EMA) between March 2000 and March 2018, only 56% were recommended by the United Kingdom (UK)'s National Institute for Health and Care Excellence (NICE) for reimbursement (14). Moreover, less than half of new cancer medications assessed during 2013–2017 across 20 countries received positive reimbursement recommendations (15). While market dynamics (such as the availability of cheaper or clinically superior therapeutic options) could influence these trends, it is anticipated that greater interaction/alignment between different stakeholders could improve efficiency in the drug development processes and increase the availability and access to innovative therapies to improve patient outcomes (16).

**Table 1:** Characteristics of different agencies (2, 10, 11).

	**Regulatory approval**	**HTA assessment (to inform reimbursement decisions)**
Legal mandate	Usually defined within national public health legislation, with regulatory bodies accountable to the government in their jurisdiction.	HTA may be undertaken by a group within and accountable to a payer, and/or by groups within and accountable to a government department, university, hospital, research institute, or industry.The coverage body (payer) is usually specified within the rules and regulations of the healthcare system in which decisions are being made and are usually accountable to the healthcare system within which they operate. In some healthcare systems, the role and responsibilities of a coverage decision-making body may be defined in legislation with accountability to government.
Primary role	Provide market authorization within the mandated jurisdiction on the basis of an assessment of safety, quality, efficacy, and risk–benefit profile	Support for clinical and coverage decisions within a particular healthcare system on the basis of assessment of relative effectiveness, costs, and in some, system affordability, value for money, and values within the system
Decision	Evaluates whether the clinical benefits for patients outweigh the risks? Should this technology be available?	Assess whether the product offers useful, appropriate (and affordable) benefits for all or a select subgroup of patients in the particular healthcare system compared to what is most commonly used in the disease area?
Assessment focus	Efficacy, safety, quality (e.g., GMP)	Effectiveness, safety, quality of life, economics, budgetary impact, social, ethical, legal, organizational
Strength of evidence	Pre-launch: Efficacy and safety from RCTs (usually placebo-controlled) Post-launch: Relative efficacy or effectiveness may be considered when reviewing product's ongoing risk–benefit profile	Pragmatic RCT*, observational studies, decision-analytic techniques (modeling)
**Characteristics of studies they prioritize**		
Validity	Internal validity	External validity
Comparator	Placebo	Active control, ideally standard of care
Endpoints	Laboratory findings and surrogate endpoints	Quality of life; final clinical “hard” outcomes such as death
Time horizon	Trial duration	Lifetime or at minimum the time needed to capture all risks and benefits of therapy

*RCT, randomized controlled trials; GMP, good manufacturing practices*.

* *Not always used*.

To date, limited reviews have examined the experiences across different markets regarding harmonization/interaction initiatives between HTA/reimbursement bodies and regulatory agencies or their impacts and challenges (3). Such an exercise is needed to improve understanding of the current landscape, identify learning opportunities, and develop insight into areas requiring improvement for effective harmonization/alignment. Thus, in the present study, we aimed to provide a synthesis of the literature regarding opportunities and outcomes of interaction/harmonization initiatives between HTA bodies and regulatory agencies. The systematic literature review was supplemented by a cross-sectional survey among European HTA bodies and regulatory agencies to provide further insight into current trends.

## Methods

### Literature Search Strategy

A systematic review of the literature was performed by searching MEDLINE, EMBASE, and the International Pharmaceutical Abstracts database from their inception up to October 21, 2019. We used two sequential search strategies to identify relevant information. In the first search strategy (Strategy A), we looked for papers related to either HTA (using the terms “health technology assessment” or “cost effectiveness” or “economic evaluation” or “economic analysis” or “cost–benefit analysis” or “cost-utility analysis”) or regulatory [using the terms “drug approval” or “pharmaceutical regulation” or “drug legislation” or “pharmaceutical administration” or “European medicines agency (EMA)” or “food and drug administration (FDA)”] decision-making processes (Supplementary Table 1). In the second strategy (Search B), we combined the keywords in Search A with “synergy” or “collaboration” or “alignment” or “partnership” “harmonization” or “scientific advice” or “parallel consultation” (Supplementary Table 2). Citations from different databases were combined in Endnote X9 (Clarivate analytics®, USA), and duplicates were removed. Subsequently, the titles and abstracts were screened, and those deemed likely to be eligible were subjected to full text assessment. Once the relevant articles were selected, additional articles were identified by exploring their bibliographies. We also searched for gray literature (working papers, commissioned reports, policy documents, etc.) via Google scholar and several national and multinational institutional websites including those of the EMA, US FDA, and the European Network for Health Technology Assessment (EUnetHTA) (Supplementary Tables 3–5). We started with general searches on websites and reviewed the hits to identify relevant materials. We then followed up to search their references and ascertain specific case examples.

### Study Inclusion Criteria

Only studies published in the English language at the time of this review were included. Moreover, to be eligible for selection, a report had to describe in whole or in part assessments on harmonization/interactions between HTA bodies and regulatory agencies, focusing on mechanisms, implementation models, outcomes, or challenges. Our review centered mainly on pharmaceuticals, although in some respects, insights from medical devices were considered. Furthermore, the review focused on reports from Europe, North America, Australia/New Zealand, and Asia. However, with the exception of Europe in which we decided *a priori* to include all relevant data regardless of country, for all others, only information from high-income countries (HICs) were targeted. An HIC was defined as per the criteria used by the World Bank to include any country with a gross national income per capita of US$12,376 or more in 2019 (17). All articles were screened by one author (ROA). However, a second author (MLDB) provided a rapid perusal of the appropriateness of inclusion of documents in the final report.

### Online Survey

An online cross-sectional survey among European HTA bodies and regulatory agencies was conducted using LimeSurvey® Pi (v3.1.4). The same set of questions seeking insight into HTA–regulatory agencies interactions were sent to both agencies (Supplementary Table 6). The survey was conducted between January and April 2020.

### Analytical Approach

We undertook a narrative synthesis that was largely inductive in nature, that is, centered on themes that were described or highlighted in detail in the literature (18). However, we also applied a deductive method by synthesizing specific information such as those related to alignment of evidentiary requirements, stakeholder involvement and perspectives, and program implementation challenges and successes. The key papers selected were those that had information to help develop the main themes for the report. However, references were also made to other papers outside those informing the themes to allow for broader contextualization. Information originating from the survey was used to corroborate the findings from the systematic review.

## Results

### Literature Search Results

**Figure 1:**
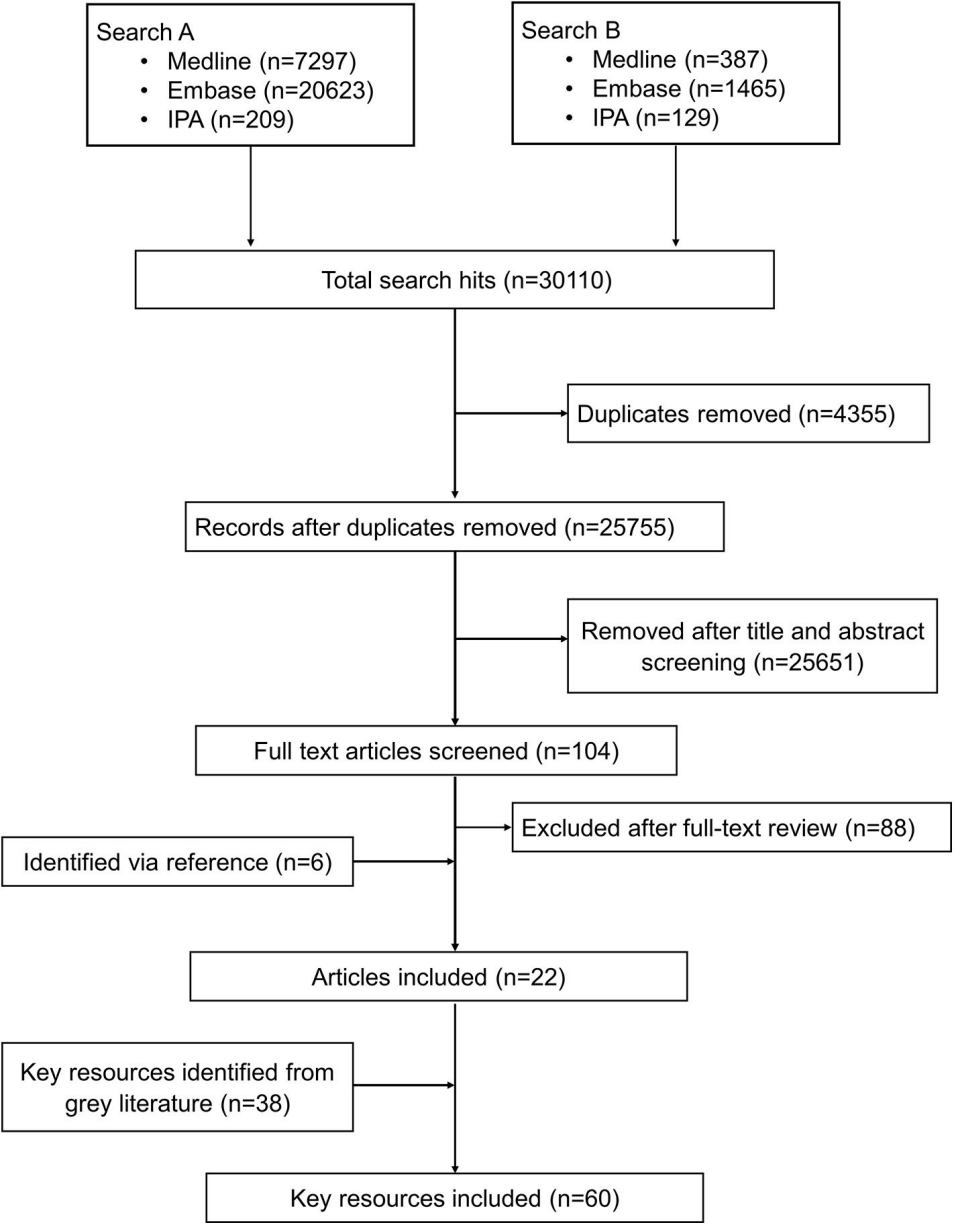
Schematic process of the review.

The bibliographic search identified 30,110 citations from which 4,354 were duplicates. Of the remaining 25,755 unique articles, 25,651 were excluded as unrelated based on titles and abstracts and 104 articles were selected for full-text evaluation. Of the 104 articles undergoing full-text assessment, 16 were retained and 88 articles were excluded. An additional 6 articles were identified by reference screening, and 38 more resources were retrieved via the gray literature search particularly from institutional websites. Figure 1 summarizes the flowchart of the reports' screening steps. The description of the key reports included in this review are provided in Supplementary Table 7.

### Survey Response

The online survey received responses from 22 HTA bodies and 6 regulatory agencies. Response rates were 18% (6/34) and 61% (22/36) for regulatory agencies and HTA bodies. The HTA bodies and regulatory agencies were from 21 and 5 different EU countries, respectively. Of the regulatory agencies, one was from Western Europe, two from Northern Europe, two from Central and Eastern Europe, and a representative from the EMA. Among the HTA bodies, nine were from Western Europe, seven from Central and Eastern Europe, four from Northern Europe, and two from Southern Europe. In total, 21 of the 32 (66%) surveyed countries (27 EU, 4 EFTA, and UK) were covered, with some variation across regions (33% Southern, 63% Northern, 70% Western, and 83% Central/Eastern Europe).

### What Is HTA/Regulatory Harmonization—Is It Necessary?

Harmonization is broadly considered to encompass the streamlining of regulatory and reimbursement processes (19). It is also deemed process oriented and centered on reducing the time between regulatory and reimbursement decisions and minimizing duplication of work (10, 20). Such an approach is viewed to have potential positive implications for the healthcare system in terms of improving patient care, innovation, and system sustainability (3, 7, 16). Regardless, there are divergent views as to whether harmonization between HTA and regulatory agencies is needed and if at all desirable. Proponents of harmonization initiatives posit that it presents an opportunity to develop economies of scale particularly with respect to evidentiary requirements and/or alignment of a product's lifecycle (11, 21). Critics, on the other hand, have highlighted that such mechanisms may have some unintended adverse impacts. In particular, separate regulatory and reimbursement functions are seen to allow health technologies to undergo robust quality assurance processes while being available on a free market (3). Thus, harmonization is recognized by some to potentially trigger overregulation that hinders the abilities for markets to function thereby leading to market failures (3). Moreover, cross-border harmonization mechanisms are also viewed by some as having the potential to diminish local decision-making power that could lead to the adoption of methods and standards that may not be well suited to the local context (22).

### Types of Harmonization/Interactions

From the survey, 67% (4/6) of regulatory agencies reported that they have an established formal link for interacting with HTA bodies while the remaining 33% (2/6) indicated that their engagement is informal. Among the 22 HTA bodies that responded to the survey, half (11/22) indicated having a formal link of collaborating with regulatory agencies. Ten out of 22 indicated that their engagement with regulators is only informal or sporadic, whereas one HTA body reported no interactions at all.

**Figure 2:**
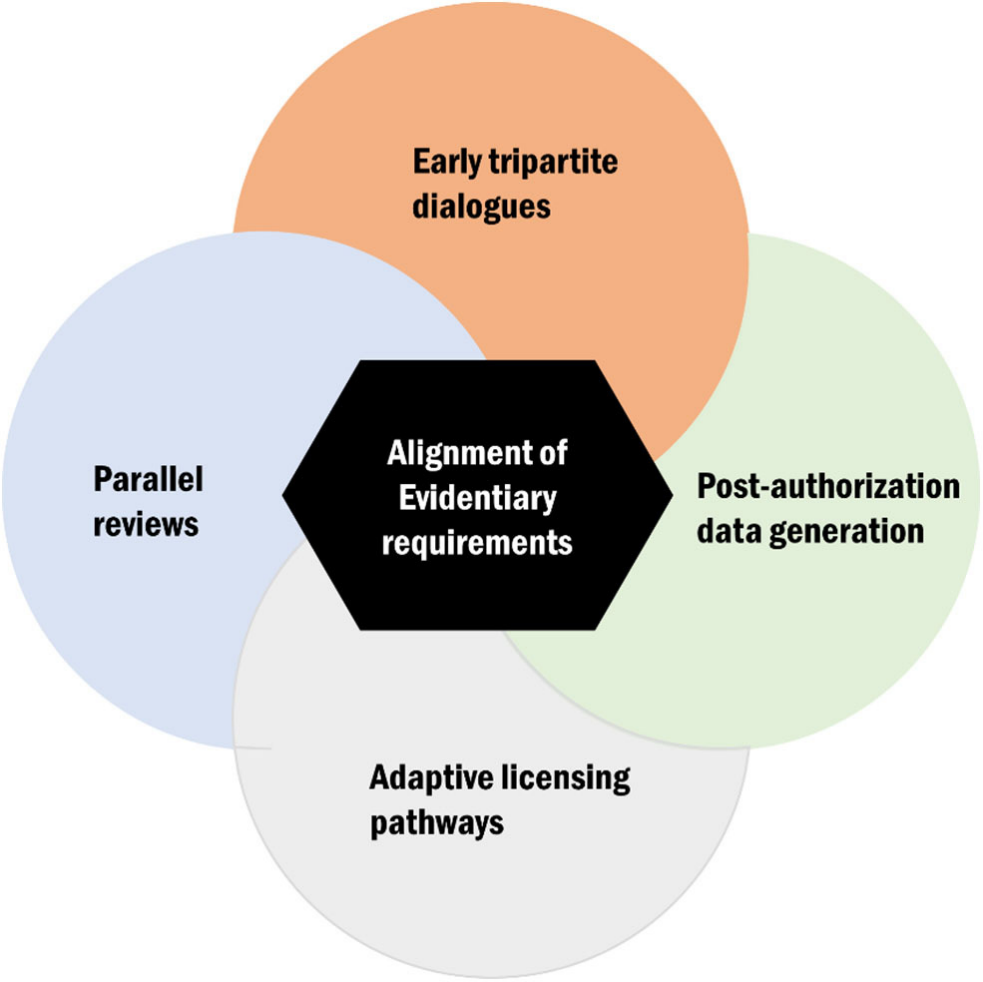
Conceptual display of the key avenues for health technology assessment (HTA)–regulatory harmonization of which alignment of evidentiary requirement is a central theme.

The interactions between regulatory and HTA agencies can be viewed across all the three phases of the product life cycle: (a) the premarketing phase, (b) the phase of actual market entry, and (c) the postlaunch phase (19). While this distinction is useful, a continuous link between the different phases is assumed. Moreover, current harmonization models or approaches can broadly be considered along the broader spectrum of evidentiary needs and those focusing on specific processes and timeframes (Figure 2). In the subsequent sections, we will discuss key issues identified from the literature and survey pertaining to harmonization mechanisms with an emphasis on alignment of evidentiary requirements, tripartite dialogues, parallel submissions (reviews), adaptive licensing pathways (3), and postmarketing collaborations and highlight their implementation challenges and successes.

### Alignment of Evidentiary Requirements

One overarching theme that transcended across the literature and survey is the need to align evidentiary requirements for HTA bodies and regulatory agencies (2, 7, 10, 21, 23). In general, while there are distinct data needs for HTA bodies and regulatory agencies, there is considerable scope to minimize the gap in their evidentiary requirements through improved alignment (24, 25). In particular, subject to demographic, epidemiological, and other factors, clinical data are generally regarded as transferable across geographical and social boundaries (22). In this context, most discussion have centered on “safety” and “clinical outcomes,” as these requirements are common to both regulators and HTA bodies (2). The discussions in the literature have given a significant boost to comparative-effectiveness research (CER) and relative efficacy (26–28). Relative efficacy is considered as the degree to which an intervention does more good than harm, under ideal circumstances, compared with one or more alternatives in achieving the desired results (26, 29).

The information obtained from relative efficacy studies has the potential to meet the evidentiary requirements of both HTA and regulatory agencies (2, 30). For example, relative efficacy studies can provide the comparative clinical data necessary to support health economic modeling or cost-effectiveness analysis. Nonetheless, while it is generally viewed that relative efficacy of a health technology will be consistent across different settings, very few studies have examined that assumption (31). In the RE-LY trial, for example, the relative efficacy of dabigatran—a new oral direct thrombin inhibitor—varied between countries even under RCT conditions, depending on the efficiency of warfarin management (32). Thus, relative efficacy can differ between different settings (countries) when healthcare practice varies. This raises additional challenge of the acceptability of evidence generated from relative efficacy studies by HTA bodies given their preference for evidence derived in their local clinical context, that is, real world settings (31, 33). Moreover, to fully meet the needs of both regulators and HTA bodies, a number of methodological issues need to be addressed. These include study design (e.g., will consideration be given to indirect comparison, or will head-to-head clinical trials between the new product and its comparator be required?) (3), endpoints (e.g., will HTA bodies consider surrogate endpoints or will they accept only clinical endpoints?), comparator (e.g., will it be the standard of care or any suitable therapeutic alternative?) (25), and target patient population (as relative efficacy varies across patient subpopulations, what will be the optimal patient population?) (2, 34).

There is increasing recognition that CER (which is very similar to relative effectiveness) can be integrated into the existing two-stage assessment framework of regulatory and HTA agencies (2, 3). However, historically, the adoption of active-comparator relative efficacy studies to support regulatory approval has been slow, as enabling laws such as the US Food Drug and Cosmetic Act of 1938, as subsequently amended in 1962, do not require assessment of comparative effectiveness (35). Regardless, in the USA, recent developments such as the establishment of the Patient-Centered Outcomes Research Institute, as part of the Affordable Care Act, has embodied a need for CER (36, 37). The most prominent drivers of CER appear to be “cost pressure” or a “search for value” (38).

From the survey, one respondent indicated that, in 2018, a proposal from their government suggested that the regulatory agency should make the evaluations on relative efficacy proactively to inform the reimbursement decisions by the national HTA body. It was noted that several objections were raised to this proposal and that this suggestion has not yet become a formal regulated duty of the regulatory agency. Regardless, the summaries of new drugs published nationally by the regulatory agency have since then been slightly modified to include more information on relative efficacy.

In general, a standard methodology for compiling comparative data is yet to be developed (39). Thus, there exist ambiguity as to how CER will be appropriately designed to reflect regulators, HTA bodies (or payers), patients, and clinicians' perspectives. As opined by Woodcock (36) from a regulator's perspective, “the tolerance for (and recognition of the probability of) error is probably the greatest divide separating the CER enterprise and the current framework for medical product regulation.”

Global initiatives such as the Green Park Collaborative is exploring the scientific feasibility of developing methodological guidance relative to evidence generation that meets the needs of different stakeholders (40). In Europe, following the publication of the conclusions of the Pharmaceutical forum in 2008, the European commission gave the EMA the political mandate to interact with HTA bodies with the aim to improve the availability and best use of data relevant to HTA (41). The primary objective of this joint project of regulators and HTA bodies was to examine how information in the European Public Assessment Reports (EPARs) can contribute to relative effectiveness assessment by EU Member States' HTA organizations (EUnetHTA). The collaboration between the EMA and EUnetHTA on EPARs started in February 2010 and lasted for more than 2 years. An evaluation of the EPAR pilot program suggested that it provided a platform for discussions about better exchange of data and information (42). It was further noted that the parallel review of EPARs has been helpful to the individual organizations to not only critically review the end-product “assessment report” employing a predefined methodology but also mutually identify areas for future improvement (42). Consequently, the EMA's Road Map to 2015 identified the need for further improvement of EPARs given their use for HTAs (12). A joint EMA-EUnetHTA 3-year workplan 2013–2015 was further instituted to build on this work (43). A report of this 3-year work plan was published in March 2016, which suggested that it facilitated the identification of areas for possible synergies as well as helping to improve understanding of the differences between individual agencies' procedures (44).

**Figure 3:**
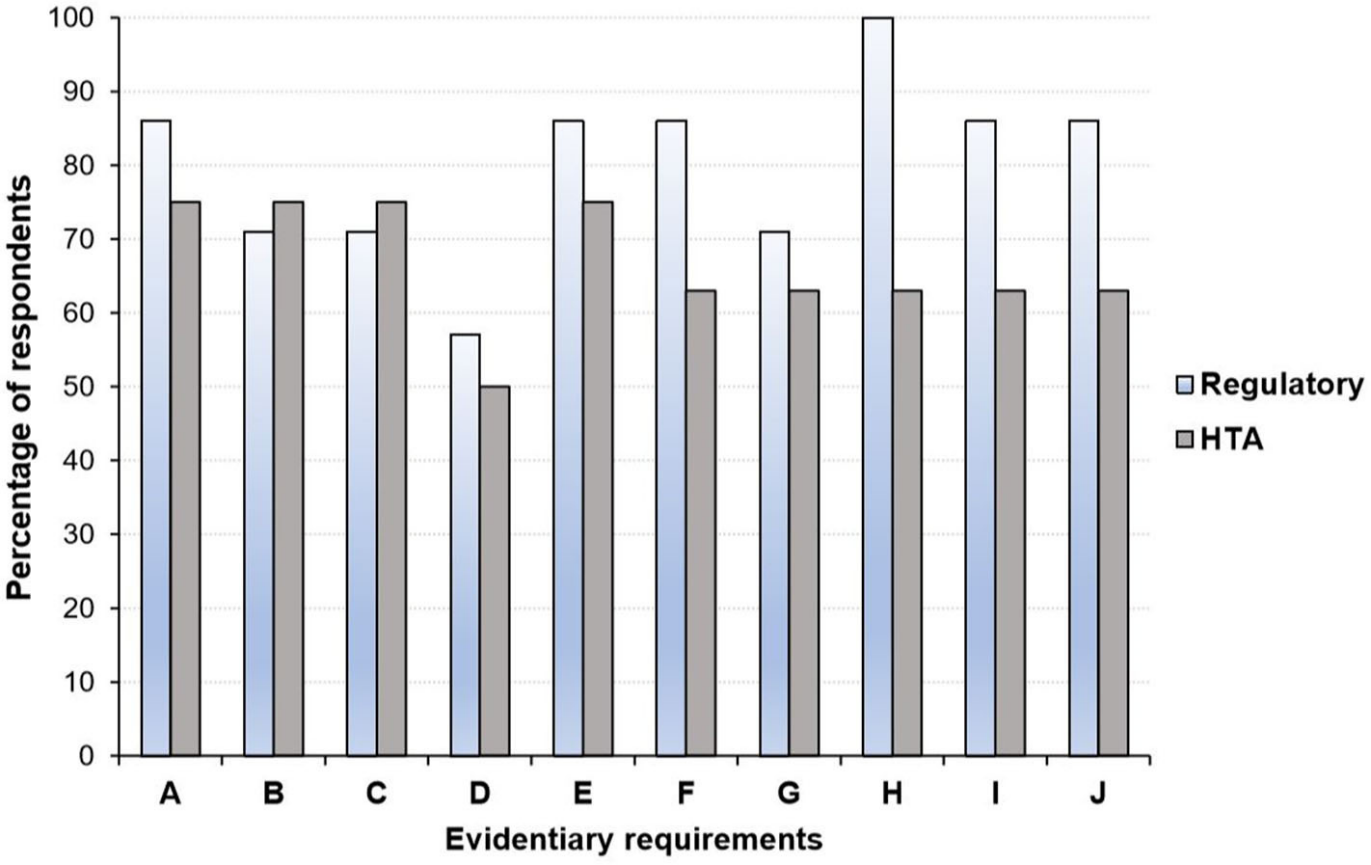
Perspective of health technology assessment (HTA) assessors and regulators regarding areas where alignment in evidentiary requirement could occur*. **(A)** Acceptable primary end point. **(B)** Inclusion of active comparator arm in the trial. **(C)** Use of patient reported outcomes. **(D)** Use of health-related quality of life measures. **(E)** Choice and use of surrogate measures. **(F)** Criteria considered in choice of comparator: therapeutic. **(G)** Use of subgroup analyses. **(H)** Inclusion and choice of secondary efficacy parameters. **(I)** Definition of unmet medical need. **(J)** Use of biomarkers to monitor patient outcomes. HTA, health technology assessment. *Graph produced by author using data from Wang et al. (25) in which a questionnaire-based survey was conducted among regulators (*n* = 7) and HTA agencies (*n* = 8) between August and September 2016.

Overall, there is some recognition that the gap in evidentiary requirements between regulatory and HTA agencies has narrowed over the past few years (25, 45, 46). For example, Dekker et al. (46), recently examined the similarities and differences in evidentiary requirements of regulatory agencies and HTA bodies (in particular, NICE) with respect to Alzheimer's disease approved. They found a large overlap in the inclusion of phase III RCTs in regulatory and HTA assessments, although the focus on specific outcomes differed slightly (46). Moreover, a 2016 survey revealed close alignment of the perspectives of HTA bodies and regulators on several evidentiary blocks (Figure 3) including the use of patient reported outcomes (PROs), whereas disagreements in areas such as the inclusion of secondary efficacy parameters were documented (25).

### Tripartite Early Dialogues

In several markets, systems exist for pharmaceutical manufacturers to seek advice from regulators during the design of their clinical development programs (47, 48). Although the advice provided through these so-called scientific advice procedures is usually not legally binding (48), adherence to the recommendations can minimize the risk that regulators will later raise objections during assessment of the corresponding marketing authorization applications. For example, an analysis by Hofer et al. revealed that from 2008 to 2012, 85% of applications that received and followed early scientific advice by the EMA were ultimately granted marketing authorization compared to only 41% that did not (49). The concept of early dialogue with HTA bodies is relatively new (50). Regardless, this form of engagement offers manufacturers an opportunity to obtain early insight regarding the evidence needs (e.g., safety, efficacy/effectiveness, cost effectiveness, budgetary impact) and how this should be communicated to reduce bottlenecks during product launch. From the perspectives of the regulatory and HTA agencies, early engagement with developers has the potential to improve the efficiency of the decision-making process (11, 51).

Harmonizing this process via tripartite “early dialogue” meetings consisting of regulators, manufacturers, and HTA bodies can increase collaboration and improve understanding among the different parties. Regulatory agencies and HTA bodies can offer joint (parallel) advice (in areas such as defining unmet medical need, analysis methodology, acceptable primary endpoints, etc.) (25) and discuss divergent data needs with the aim of minimizing discrepancies and identifying trade-offs, whereas manufacturers can have a single forum to discuss any potential claims or concerns (52). The opportunity to incorporate patients' and clinicians' perspectives in these discussions could further enrich the data needs (11, 53).

Tripartite advisory models have been implemented in different jurisdictions (Table 2). In 2010, the EMA commenced a pilot on Parallel Scientific Advice (PSA) together with HTA bodies (44). Moreover, in May 2014, the EMA released a “Best Practice Guidance for Pilot EMA-HTA Parallel Scientific Advice Procedures” for public consultation (63), and the EMA-HTA PSA was formalized in 2015. Some of the issues specified in the guidance document were the following: (1) all medicinal products are eligible irrespective of their eligibility for the central procedure; (2) it is the applicant's choice which HTA bodies could participate (usually the number of HTA bodies participating should not exceed 5); (3) the invited HTA bodies are not obliged to participate; (4) a common briefing document is used; (5) advice is not legally binding (however, when regulators give scientific advice, it is based on the current state of the art in medicines development). While they recognize that due to evolving scientific knowledge, an alternative approach to that advice may be appropriate; where companies choose not to apply the advice, they are requested to justify clearly their position in any subsequent marketing authorization application) (52); (6) the process is confidential; and (7) the Administrative work is done by the EMA (63). As of December 2015, the total number of completed procedures for the EMA-HTA scientific advice was 63 (44). An analysis of 43 PSA procedures showed that the HTA bodies with highest representation were NICE (involved in 90% of all PSA procedures), followed by the German Federal Joint Committee (65%), Italian Medicines Agency (45%), Dental and Pharmaceutical Benefits Agency (Sweden) (35%), National Authority for Health (France) (19%), Main Association of Austrian Social Security Institutions (10%), Catalan Agency for Health Quality and Assessment (10%), and National Institute for Sickness and Invalidity Insurance (Belgium) (3%) (45).

**Table 2:** Overview of early tripartite dialogues and parallel submission interactions.

**Region/country**	**Stakeholders**	**Name of program**
Australia	TGA (regulator) PBS (HTA user/payer)	Scientific advice on development (pilot) (54)
Australia	TGA (regulator) PBAC (HTA user/payer)	Parallel submission/review (55)
Canada	Health Canada (regulator) CADTH (HTA user/payer)	Parallel submission/review (56)
England and Wales	MHRA (regulator) NICE (HTA user/payer)	NICE Scientific Advice Programme (57)
Sweden	MPA (regulator) TLV (HTA user/payer)	Scientific advice on development (58)
The Netherlands	MEB (regulator) ZIN (HTA user/payer)	Parallel submission/review (Pilot) (59)
Europe	EMA (regulator) EUnetHTA (multinational HTA network)	Parallel consultation (23)
Europe	Multiple stakeholders, including EMA, MHRA, MPA, BfArM, AFSSAPS, AIFA (regulators) NICE, TLV, G-BA, CEPS, AIFA (HTA user/payer) EUNetHTA (as observer) FDA (as liaison)	Tapesty Network (Scientific advice on development) (60)
US	FDA (regulator) CMS (HTA user/payer)	Parallel submission/review (61, 62)
Global	Multiple stakeholders	Green park collaborative (scientific advice on development) (40)

*TGA, Therapeutic Goods Administration; PBS, Pharmaceutical Benefits Scheme; PBAC, Pharmaceutical Benefits Advisory Committee; CADTH, Canadian Agency for Drugs and Technologies in Health; MHRA, Medicines and Healthcare products Regulatory Agency; NICE, The National Institute for Health and Care Excellence; MPA, Medical Products Agency; TLV, The Swedish Dental and Pharmaceutical Benefits Agency (Tandvårds- och läkemedelsförmånsverket); MEB, Medicines Evaluation Board; ZIN, The National Health Care Institute (Zorginstituut Nederland); EMA, European Medicines Agency; FDA, Food and Drug Administration; AIFA, Italian Medicines Agency (L'Agenzia Italiana del Farmaco); BfArM, The Federal Institute for Drugs and Medical Devices (Bundesinstitut für Arzneimittel und Medizinprodukte); CMS, Centers for Medicare & Medicaid Services*.

An analysis of the first 11 EMA-HTA PSAs also showed that the majority of the questions posed by developers were in relation to the design of clinical studies such as endpoint and comparators (Supplementary Figure 1) (64), a trend that is expected. Moreover, analysis of 518 answers provided by regulators and HTA assessors in 31 PSAs conducted during 2010–2015 revealed that full agreements, partial agreements, and disagreements were reached in 61, 23, and 16% of responses, respectively (45). In particular, the occurrence of divergence in recommendations provided were seen to be lowest for the study patient population and highest regarding selection of comparator (45). Where divergence in recommendations have occurred, notable cases of successful compromises in product development have also been reported from parallel EMA-HTA PSAs. For example, in one instance, a company preparing to launch a novel therapy for chronic obstructive pulmonary disease (COPD) proposed utilizing a licensed comparator in its pivotal trial. The EMA sided with this proposal; however, an HTA representative who was present requested a different comparator not licensed for use, yet routinely used. The solution was to introduce a new arm of the pivotal study to include both comparators, meeting the recommendations from both advisors (65). In another case, a pharmaceutical company had developed a novel therapy as a first-in-class treatment for a rare oncological disease. Since no other product had previously been licensed for this indication, the company proposed standard of care as its comparator, and the EMA agreed. However, HTA bodies present requested the use of an off-label active comparator and the pharmaceutical company settled on this pathway (53).

When Tafuri and colleagues (66) analyzed the uptake of the comparator recommendations at the time of 31 PSAs (during 2010–2015) within the actual development, they found that manufacturers implemented comparators to address both the needs of regulators and of at least one HTA body in 12 out of 21 studies (almost 60%). Studies in which manufacturers followed the regulators' and >50% of the HTA bodies' advice were 8/21 (38%), while those following exclusively the regulatory advice were 7/21 (about 30%). Only in two studies did the manufacturer implement recommendations, neither from the regulators nor from the HTA advice. Moreover, it was found that changes were never implemented solely based on the HTA advice. For the primary endpoint in all included studies (23 out of 23) manufacturers implemented both the requests of the regulators and at least one HTA body. In 15 studies out of those 23, the manufacturer complied with the advice of both the regulators and >50% of the HTA bodies. These data suggest, to some extent, that manufacturers seem to be more inclined to satisfy the regulatory advice (66). In 2017, the EMA-HTA PSA was replaced with the EMA-HTA Parallel Consultation (PC) process, the key update being the incorporation of the European Network for Health Technology Assessment (EUnetHTA) and Early Dialogue Working Party (EDWP), although all other aspects of the PSA remained largely unchanged (23).

Overall, regulators and HTA bodies have expressed positive views about tripartite dialogues (11, 51, 52). Moreover, pharmaceutical manufacturers have identified several benefits with the process including reducing development program risk and creating common multistakeholder understanding of unmet medical need and acceptability criteria for innovative study design approaches (53). Nonetheless, the value of any dialogue is dependent on the stability of the advice or when it is provided. Thus, to derive maximum benefit, planning is critical. For example, if advice is sought too early, issues that may arise after the clinical trials have commenced may not be addressed or it would be costly to revise and collect new information (65). On the other hand, if sought too late, there may be insufficient time to complete the clinical trials before the target market date. For a potential product that is not a strong candidate for an early access pathway, it is suggested that the ideal time for a sponsor to initiate the dialogue is during phase II evaluation, or just after achieving proof of concept, and at least 6 months prior to planned phase III initiation (53). Regardless, earlier consideration of strategic advice in the phase I setting may be necessary to discuss assumptions and concepts for potential accelerated development opportunities before data generation. Overall, increased confidence in the PSA dialogue process is likely to be achieved via provision of formal (e.g., written) feedback (3). However, projects have tended to adopt different approaches. For example, whereas NICE-MHRA PSA Program (57) and the Tapestry Network pilots (60) provide formal written postconsultation reports, in the case of the Swedish authorities, the responsibility for documenting any discussions lies with the applicants themselves (3). It is also useful for manufacturers pursuing tripartite dialogue to recognize that any advice provided is contextualized within existing knowledge, and this may evolve as scientific and clinical understanding progresses.

Despite the potential benefits of early tripartite dialogues, some perceived disadvantages of the process include the fact that there are no formal mechanisms for addressing divergence between the parties. Moreover, the desire to achieve one consolidated position from HTA bodies and regulatory agencies has been opposed in some settings, as this is viewed to limit the rights of individual agencies to develop and express their own independent views (54). Another potential setback with tripartite meetings is that they may present additional financial hurdles for pharmaceutical companies, as these dialogues are usually provided as a fee-for-service (23, 57). Regulatory capture could also be seen to present a conflict of interest as early interactions can imply that regulatory and HTA bodies are potentially engaged in codevelopment of medicines (65). Furthermore, there are concerns that the role of HTA agencies as final gatekeepers may be compromised through early involvement with developers. However, according to McAuslane et al., if the current challenges with early dialogue are properly addressed, it “is the process that will likely provide the greatest return on investment of time and effort to identify, develop, review, and recommend important new medicines, especially those that address an unmet medical need” (67).

### Parallel Submission (Review)

Parallel submissions seek to reduce the time between regulatory and reimbursement decisions by aligning their review processes. In Australia, as part of a Memorandum of Understanding, a parallel process of regulatory [Therapeutic Goods Administration (TGA)] and reimbursement [through Pharmaceutical Benefits Advisory Committee (PBAC)] submission has been implemented since 2011 (Table 2) (68). While a product must still be listed on the Australian Register of Therapeutic Goods before it is listed on the Pharmaceutical Benefits Scheme (PBS), the Australian Drug Evaluation Committee (ADEC) and (PBAC) can now receive submissions for a product in parallel. A PBS listing cannot occur prior to the product being listed on the Australian Register of Therapeutic Goods for the relevant indication. Moreover, if the final TGA approval for a product is received before a PBAC recommendation has been made, the PBAC secretariat will check that any proposed PBS listing is fully consistent with the final TGA registration (55). If there are any discrepancies, the PBAC reconsiders its evaluation. By 2012, five products (linagliptin, testosterone solution, ivabradine, mycophenalate sodium, and rifaximin) had their PBAC decisions deferred until ADEC recommendations were made (69). Analysis of regulatory and HTA review found that when TGA took a longer than average time to review products, those products typically received a negative recommendation from PBAC, although it was unclear whether similar issues were raised by both agencies (70). The usefulness of parallel submission has been highlighted in one case study involving pembrolizumab—a medicine used to treat melanoma that has spread or cannot be removed by surgery (advanced melanoma) or to prevent postsurgery relapse. The manufacturer put the medicine through parallel review. This resulted in the listing of pembrolizumab on the PBS, only 4.5 months after TGA approval (71). Ordinarily, when undertaken sequentially, the median time between a positive TGA recommendation and PBS listing has been found to exceed 30 months (69). An analysis by the Center for Innovation in Regulatory Science (CIRS) on the appraisals of 38 New Active Substances (NASs) introduced between 2014 and 2018 suggested that the TGA/PBAC parallel process may have been instrumental in the shorter time between regulatory approval to HTA decision in Australia (median = 17 days) compared to Canada (median = 161 days), France (median = 202 days), England (median = 311 days), Germany (median = 123 days), Scotland (median = 173 days), and Sweden (median = 158 days) (56).

In Canada, a manufacturer can submit for a Canadian Agency for Drugs and Technologies in Health (CADTH) Common Drug Review before a Health Canada Notice of Compliance (NOC) is issued. For the Health Canada/CADTH parallel review process, the submission to CADTH can occur up to 180 days before the date of anticipated NOC from Health Canada (72). This accelerates the process since the Canadian Drug Expert Committee can release its reimbursement recommendation to CDR immediately following the regulatory decision. A review of 56 NASs appraised by CADTH from 2014 to 2016 showed that parallel review reduced the time from regulatory approval to HTA recommendation. The median time from Health Canada approval to CADTH recommendation was 158 days for drugs undergoing parallel review (*n* = 22) compared to 377 days for drugs undergoing sequential review (*n* = 34) (73).

In 2010, the US Food and Drug Administration (FDA) and Centers for Medicare and Medicaid Services (CMS) announced the FDA-CMS Parallel Review pilot program for medical devices (61). Through the program, manufacturers can request initiation of a CMS national coverage determination (NCD) while the product is still under FDA review. After 5 years, the program's impact was deemed to have been minimal, and interest among manufacturers remained low (74). In particular, only one device (Exact Sciences' Cologuard test, a multitarget stool DNA test developed for noninvasive screening for colorectal cancer) was approved through the process. The time from premarket approval submission to the final NCD was 489 days compared to an average of 612 days for other NCDs issued in 2013 (75). While this suggested that parallel-review process could shorten the expected interval from regulatory approval to coverage determination, the experience with Cologuard was only a single case. The only other product known to have undergone the pilot parallel review was Medtronic's Symplicity renal denervation system. However, the process was not completed as the device's phase III trial (SYMPLICITY HTN-3) failed to meet its primary efficacy endpoint (76). In 2016, the FDA-CMS Parallel Review was fully implemented and extended indefinitely (62). Since then, increased interest in the parallel review program among developers has been reported, and in 2017, Foundation Medicine's FoundationOne CDx next generation sequencing (NGS)-based test was approved under the scheme (77).

In May 2019, the Medicines Evaluation Board (MEB) and the Netherlands Healthcare Institute (ZIN) launched their pilot “Parallel Procedures MEB-ZIN.” The stated objective is to “shorten the time from registration to reimbursement of a medicine” (59). The “Parallel Procedures MEB-ZIN” will commence in mid-2020. However, by March 2020, two manufacturers [Insmed BV for their amikacin liposomal inhalation suspension (ALIS) (Arikayce®) and Novo Nordisk BV for their oral dosage form of Semaglutide (Rybelsus®)] had registered their products to undergo the parallel review process (59).

Overall, one major challenge with parallel review is that if a product fails to obtain regulatory approval, it renders the work of HTA bodies redundant and a waste of time and resources. In Australia, if regulatory approval is not granted for a product that goes through parallel review, the sponsor company is made to pay a cost-recovery fee to compensate for the resource used for HTA evaluation (70). This may not be ideal for developers. Thus, to minimize such an occurrence, it may be useful that the HTA's initial review centers on the less resource-intensive components so that the time between assessments remains shortened while at the same time not committing too many resources in the event of negative regulatory outcome. Moreover, the institution of a mechanism to select technologies that are most likely to secure regulatory approval to undergo parallel review might also be essential. Regardless, without the requisite data to meet the evidentiary needs of regulators and HTA bodies, parallel submission may not always lead to earlier market access, as an unfavorable review outcome could still occur (3). Hence, a strategy of combining alignment of evidentiary data needs and early dialogue may be necessary to ensure that trials are designed in such a way that the data necessary to meet the needs of both agencies are collected. One such initiative is the EXCITE Programme by The MaRS Excellence in Clinical Innovation and Technology Evaluation, which brings together a broad spectrum of research under one harmonized platform based on relationships brokered with academic health research facilities across the Ontario province in Canada. Through EXCITE, medical devices undergo a combination of clinical testing and HTA review in order to obtain the evidence needed for both federal licensing and provincial health system adoption (78).

### Adaptive Licensing Pathways

Since the emergence of drug regulation, approval mechanisms have been challenged by the need to achieve a balance between ensuring timely access for patients without compromising safety (79, 80). The traditional regulatory paradigm is characterized by well-defined structures and rigid processes that require several years of research, development, and authorization for a medicine to reach the market (81). However, this mechanism is criticized as outdated and that it ignores the complexities of health technologies, as well as the diversity in population features and disease progression (3). Hence, there has been a push for a shift from the traditional approach, which relies on extensive testing and the marketing authorization for large groups of patients (with a single decision point focus) to a procedure that employs periodic or staged assessment and reassessment using an evolving evidence base (81, 82). There have been a number of proposals (Supplementary Table 8) advocating for planned adaptive approaches to drug licensing using terms such as “staggered entry,” “adaptive approval,” and “progressive authorization.” However, much of the conceptual framework of adaptive licensing emanated from the New Drug Development Paradigms (NEWDIGS) collaboration that started in 2010 as an initiative of the Massachusetts Institute of Technology (MIT) and was hosted by the MIT Center for Biomedical Innovation (81). The concept has, however, been renamed to “adaptive pathways (APs)” to better reflect a focus on the development and managed introduction of medicines rather than a new way of regulating and authorizing medicines (83). Moreover, over the past years, regulators in different jurisdictions have had several mechanisms in place to facilitate an earlier access of new promising medicines especially in areas of high unmet medical needs and for orphan diseases (84). In Europe, the EMA early access and registration tools include Priority Medicine (PRIME) (85), conditional marketing, and authorization and approval under exceptional circumstances (86), as well as compassionate use exist (87). Hence, the concept of AP is not necessarily a new licensing pathway but a way of getting clinical data in order to design a smart development program to meet the evidentiary needs (82, 83).

In 2014, the EMA launched the AP pilot program inviting participation from companies that had candidate products that were early in clinical development (88). The AP is based on key principles such as the need for early dialogue (collaboration) with multiple stakeholders (regulators, HTA agencies, and patient and healthcare professional representatives) to identify a subset of patients expected to present a favorable benefit–risk profile as well as significant emphasis on the use of real-world data to supplement clinical trial data. Other criteria for the selection of products for the APs pilot was an iterative development plan. The iterative development plan can follow two registration scenarios: (1) starting with a marketing authorization for a well-defined subpopulation and expanding the population (“widening of the indication” scenario) or (2) obtaining a Conditional Marketing Authorization, whether based on surrogate endpoints or not and conducting confirmatory studies afterwards (“prospectively planned reduction of uncertainty” scenario) (Supplementary Figure 2) (89). In the AP pilot project, EMA received 62 applications out of which seven progressed to a formal scientific advice (one) or parallel regulatory-HTA scientific advice (six). The reasons for non-acceptance into the pilot included (1) development programs that did not have scope for expansion and iteration, (2) proposals for areas without unmet need, and (3) late stage development programs (where no changes to the plan could be effected) (88).

In general, the “safe harbor” environment of APs is intended to foster an increasing willingness to share information, data, and expertise, thereby improving collaboration between the different agencies (83, 89). For example, actual and modeled clinical development and licensing programs of three case studies as part of the Janus initiative concluded that the adaptive licensing approach increases stakeholder commitment (90). Regardless, to achieve the intended acceleration in patients access, the APs need to identify ways to reduce the time lag between marketing authorization and reimbursement. This further reinforces the importance of early dialogue with HTA bodies and regulatory agencies as well as alignment of evidentiary needs (53). To facilitate such interactions, the Innovative Medicines Initiative (IMI) through the ADAPT-SMART initiative assembled together stakeholders to develop better ways to achieve APs (91).

To ensure efficient adoption of APs, appropriate legal structures need to be in place (80). For example, a report by Oye et al. indicated that attorneys from the US FDA, EMA, and the Singapore Health Sciences Agency found that existing statutes in their jurisdictions provided authority for adaptive licensing, although gaps were noted in the Canadian legislation (92). Moreover, the success of APs requires a more “system-wide” approach including, for example, the willingness of patients to participate in clinical research to evaluate benefit/risk and determine if new medicines were effective and how different stakeholder perspectives are reflected in the decision-making process (90). As highlighted by Schulthess et al. (93), the bigger challenge facing the adoption of APs borders on how to incorporate different decision-making processes into AP methodologies “to ensure the appropriate balance is struck between earlier access to new medicines, a given regulator's willingness to facilitate that to occur, a healthcare provider's willingness to accept more focused data before prescribing a new medicine, as well as the provider's correlating willingness to restrict their off-label prescribing practices and to participate in real-world clinical research to progressively reduce uncertainties, a given payer's willingness to purchase such medicines, and having strong multifaceted postauthorization systems in place to facilitate all of this in as safe and dependable a manner as possible.”

### Post-authorization Data/Evidence Generation

At the time of marketing authorization, the available information relating to a medicine may not yet be sufficient to fully assess the benefit/risk profile to the desired degree of certainty (94, 95). Therefore, regulatory agencies may require the generation of additional data, for example, in the form of clinical studies after authorization. Nonetheless, there is considerable opportunity for HTA bodies and regulatory agencies to collaborate toward providing guidance on the design of postapproval studies that can fulfill both of their needs (19, 25). This would be necessary to avoid developers' duplication of efforts in the postlaunch evidence generation phase, for example, with respect to the planning and execution of postauthorization efficacy studies (PAESs) and postauthorization safety studies (PASS) (44). In this context, a collaboration between EMA and EUnetHTA on postauthorization data collection commenced in March 2011. The discussions began from collaboration on two projects: European Network of Centers for Pharmacoepidemiology and Pharmacovigilance (ENCePP), under the leadership of EMA, and the EVIDENT database—a database containing evidence information on new technologies. Through this collaboration, reports on the feasibility of conducting postapproval studies in Europe were produced as well as guidelines on the necessary methodological standards to execute such studies (96). In 2016, the EMA instituted the Scientific Advice Working Party (SAWP)/The Pharmacovigilance Risk Assessment Committee (PRAC) joint scientific advice for the PASS/PAES studies (97).

There are additional opportunities for HTA bodies and regulatory agencies engagement around optimization of real-world data (RWD) generation such as the use of patient registries (98) or the opportunity to share periodic benefit–risk assessment reports and therapeutic value reassessments. This would include alignment on key areas such as outlining the definition of data to be collected (i.e., minimum dataset) in registries (19). In this context, the EMA launched an initiative with EUnetHTA representation in 2015 to facilitate the establishment of patient registries as well as introducing and supporting a systematic and standardized approach to their contribution to serve regulatory and HTA needs (99). There is also opportunity for collaboration regarding the use of additional sources for the collection of RWD, such as data derived from electronic patient records. Within this space, the IMI GetReal project (2013–2016) was a multistakeholder initiative, involving regulators, HTA agencies, patient organizations, academics, and industry that sought to propose and create tools to support new robust methods of RWE synthesis for use in medicine development and decision-making throughout the product cycle including the initial regulatory and postapproval phases. While this project has ended, the work continues via the IMI GetReal initiative launched in 2018 (100).

### Barriers and Challenges to Harmonization

Despite the increasing interest and potential for synergies between HTA bodies and regulatory agencies, several barriers, hurdles, and challenges were identified via the survey and systematic review. Some survey respondents indicated that the intensity of cooperation is low, as there is no institutional framework for cooperation and it all comes down to individual initiatives. Moreover, there is a need to build trust and understanding between all relevant stakeholders including HTA bodies, regulatory agencies, payers, manufactures, clinicians, and patients through effective communication and transparency (11, 51). In particular, mechanisms for continuous open dialogue are important to foster the development of stronger relationships and minimize misconceptions. For example, some manufacturers have expressed misgivings about harmonization initiatives for fear that HTA bodies/payers might be able to influence market authorization decisions and vice versa (51). Improved knowledge of each other's functions, roles, and remits may also reduce misunderstandings that may lead to unintended policy consequences that can create misalignment (101).

Furthermore, greater understanding of each agency's remits and processes provides a medium for conveying realistic expectations about the extent of coordination and agreement that is achievable. An HTA body responded in the survey that it is sometimes “difficult to separate evidence review from policy issues and financial consideration” when engaging with regulators. There are also practical differences in areas such as evidentiary requirements that need to be acknowledged. For example, HTA bodies may be hesitant to accept trials using placebo control when an active comparator exists, although this may be acceptable to regulators (2, 3, 25). On the other hand, it is conceivable that payers and HTA bodies may agree to some of the clinical outcomes specified by regulators while also highlighting the need for additional data such as those related to quality-of-life and long-term effects (46). These potential differences need to be anticipated so that efforts can be channeled into areas with greatest potential for harmonization. Furthermore, it is also important to establish leadership and clearly defined roles and responsibilities to minimize a culture of blame if the intended outcomes of harmonization are not attained (101). For example, regarding the use of CER and relative efficacy studies, it is critical to outline how this should be implemented, who will be responsible, how will such research be funded (and by whom), and how will the data generated be disseminated. It must be anticipated that allocation of responsibilities can be a sensitive area, as different agencies may seek to fiercely guard their existing responsibilities and operations (54, 101).

Harmonization is also likely to introduce changes to the current regulatory and reimbursement pathways that would only be possible with the implementation of new supportive structures and, if required, legislations (80). For example, in some cases, legislative amendments may be needed since the roles of regulators are specifically defined in law or by their governments and HTA bodies may or may not be fully established in law. Indeed, one survey respondent indicated that within their jurisdiction, “HTA assessment is not mandatory and regulators are not obligate to cooperate with us.” In general, there are also concerns that regulatory structures need revamping, as they were instituted when no formal reimbursement mechanisms were in place (14). Existing laws may hinder cooperation between the respective agencies, as one survey respondent indicated that in their country, “social laws does not provide many opportunities to do so.” Moreover, the sharing of confidential information between different stakeholders may be limited by law. One respondent indicated that “although there are potential synergies there is limited scope for joint operations. Horizon scanning was explored but it was not possible for the regulatory agency to share this information.” There are also concerns among companies about how proprietary information may be handled by the different agencies given their unique working styles (101). In particular, regulators are usually bound by strict confidentiality codes, whereas payers have varied frameworks that center around transparency in decision-making (6). Thus, to support harmonization initiatives, a system is needed that offers interagency exchange of information to allow each to learn how the other is using available data to inform decision-making. For example, a central component of APs is the need for continuous data collection. In this context, information related to appropriate usage (compliance) as well as those related to effectiveness and safety collected by payers may be relevant to regulators to inform periodic regulatory assessment (3). To facilitate the sharing of data, some pilot programs have been tested as part of the EMA's “Road Map to 2015” (12). An optional information sharing process has also been established to permit Health Canada and CADTH to exchange information regarding a drug under review, for submissions filed with CADTH on a pre-NOC basis. Sponsors are encouraged to agree to this data sharing mechanism to facilitate the parallel review process (102). The FDA-CMS Parallel Review also has an inbuilt mechanism for cross-sharing relevant data (61).

Another challenge to broader harmonization is the availability of resources required for regular cooperation between the agencies. For example, one survey respondent indicated that “we are not in the same city, so face-to-face meetings need resources,” and others hinted that resources for joint training is limited. In this context, several initiatives have relied on user fees (e.g., scientific advice), and alternative funding models may be needed to ensure their sustainability (23, 57, 102). Moreover, a balance needs to be struck in the pursuit of national/regional collaborations as opposed to international harmonization initiatives. This is because while initiatives with an international scope (e.g., Green Park Collaborative) may offer high value to industry because their outputs could apply across multiple markets, and may reduce duplication of similar efforts in multiple jurisdictions, they are likely to face jurisdictional challenges as well as issues related to context-specific disparities (e.g., differences in standard of care and relevant comparators, economic and political priorities, and healthcare delivery systems).

## Discussion

### HTA–Regulatory Harmonization: Current Progress and Challenges

Improvements in health technology raise hopes for better patient outcomes and a more efficient delivery of health care. However, the processes of diffusion and implementation of new health technologies require a series of steps and engagement with different stakeholders, and many healthcare systems continue to struggle with finding ways to ensuring prompt access to safe and efficacious healthcare products for their patients. A large part of this challenge has been ascribed to the two sequential processes of regulatory and reimbursement decision-making, which is deemed to be ill-suited to facilitate timely, well-informed patient access, stimulate drug development, and simultaneously ensure routine collection and evaluation of all relevant information on benefits and risks (83). Against this background, the alignment of regulatory and HTA processes has been proposed as a means to remedy the situation toward increasing the effectiveness of decision-making, mitigating the disconnect between different agencies and their stakeholders, as well as promoting public trust in the review processes.

In this systematic review and cross-sectional survey HTA bodies, we found that there has been progress over time in narrowing the gap in evidentiary requirements between HTA bodies and regulatory agencies. Different models and approaches aimed at fostering closer interactions between agencies were also identified. The initiatives described often require organizations to work outside of their traditional remits, to engage with different stakeholders, and, in some instances, to modify their processes. Regardless, the expected level of change necessary to adopt different models or the practicalities (time and resources needed) of their implementation vary. Broadly, we have considered five key areas: early tripartite dialogues, alignment of evidentiary needs, parallel submissions (reviews), adaptive licensing pathways, and post-marketing data generation. However, these mechanisms must feedback into each other, and they should not be viewed as mutually exclusive.

Concerns over potential merger of regulatory and reimbursement functions to have adverse impacts on quality assurance processes as well as distorting the free market dynamics may explain some of the hesitance to greater collaboration between these agencies (22). Despite this, there is considerable scope to develop economies of scale particularly with respect to evidence generation. Harmonization/alignment of evidentiary requirements is likely to be feasible and most effective when targeted at common requirements that are assessed by both HTA agencies and regulators (i.e., clinical outcomes). However, greater friction is expected in areas that are more specific to each agency (e.g., economic, budgetary impact). Thus, in promoting harmonization, it is important to include outcome measures within trials that satisfy both agencies' needs. For example, regulators must ensure acceptable choice of surrogate endpoints, particularly those that have demonstrated good correlation with hard outcomes (such as mortality). Since, most HTA–regulator discussions are related to specific trial design and the selection of appropriate comparators and outcomes, a focus on product-level harmonization appears appealing to foster closer evidentiary alignment. Regardless, emphasis on initiatives addressing the evidentiary expectations of HTA bodies and regulatory agencies at the level of therapeutic areas may offer greater return at maximizing the efficient use of scarce organizational resources and for generating outputs that are of wider importance. Furthermore, significant efficiencies for both companies and the reviewing agencies may be gained via development of condition specific as well as general methodological guidance at the international level. Overall, future discussions/initiatives around alignment of evidentiary requirements for HTA bodies and regulatory agencies must recognize that integration of certain scientific elements are possible and appropriate, but others may not be suitable for alignment and that forcing such alignment could be defeatist.

Models such as APs, although still in early development and largely applied to pharmaceuticals, have potential to be adopted for medical devices as well. For example, the continued reassessment characteristics of adaptive licensing would thus align well in assessing the impact of incremental innovation and real-life usage of the medical device. Some results of parallel submission programs suggest that such mechanisms may reduce time between regulatory approval and reimbursement decisions. Moreover, some analyses have revealed the positive effect of tripartite dialogues on clinical development programs (66), although mechanisms for addressing divergence need to be clarified. Moreover, the long-term impacts of such measures are yet to be evaluated. The increasing desire for the use of real-world evidence to supplement RCT data also provides further opportunities for increased alignment between HTA and regulatory agencies throughout the product cycle, but methods and standards require further refinement with time.

A number of practical enablers, challenges, and barriers were also identified in the literature and survey, which require attention to improve harmonization between HTA and regulatory agencies. In particular, the need to build trust and buy-in from all stakeholders is important. Moreover, greater understanding of each institution's processes as well as objective characterization of each agencies' needs, responsibilities, and resources is important to building collaboration. Regardless, mechanisms need to be better developed on how to secure the exchange of confidential data between different agencies. Further consideration of resources available should inform models and approaches that are pursued. It is expected that each approach to harmonization will provide different benefits and challenges. Thus, in deciding on a harmonization approach, reflection on local contextual factors including healthcare system, political factors, and resource availability are important. While a desire for international harmonization initiatives is understandable due to their potential to generate outputs that could apply across different markets, their inherent limitation such as likelihood of lacking context-specific focus need to be recognized (22).

### Study Limitations

This study has several limitations. First, while inferences were made to specific cases, the evidence synthesis was largely qualitative and descriptive in nature, thereby amenable to interpretation bias. The included reports were mainly reviewed by a single author given the available resources and time constraints. Given the very broad nature of the topic (which resulted in substantial search hits, the majority of which were excluded), it is possible that some reports may have been missed. However, we do not think that further major themes outside of what has been discussed would have emerged. In particular, most of the themes identified from the literature were further highlighted in the survey. A number of deliberations such as PSAs are usually treated as confidential (23, 57). Thus, the information extracted from published literature may not be entirely reflective of the processes and outcomes. Thus, to gain further insight, a symposium organized by the HTx consortium (https://www.htx-h2020.eu/) has been planned for October 2020 to engage both HTA bodies and regulatory agencies on the matter of harmonization/alignment. For example, the views of different agencies on whether alignment is a more achievable goal than complete harmonization will be solicited at the forum. Furthermore, as the systematic review was limited to articles published in the English language, this may limit the generalizability of the findings. In the survey, the response rate from the regulatory agencies was low (<20%). However, the final number of included regulatory agencies (*n* = 6) is comparable to that from a 2016 survey by Wang et al. (*n* = 7) (25). Lastly, the survey respondents were mainly from Europe, and their experiences may not be generalizable to other jurisdictions.

## Conclusions

There has been progress over time in narrowing the gap in evidentiary requirements for HTA and regulatory agencies. In many European countries as well as in US, Canada, and Australia, a formal link of collaboration between HTA bodies and regulatory agencies has been instituted. To date, several mechanisms such as early tripartite dialogues, parallel submission (review), adaptive pathways to licensing, and postauthorization data generation have been explored as avenues for improving collaboration. A number of pilot initiatives have also shown positive effects of these models to reduce the time between regulatory and HTA decisions, which may translate into faster patients' access to life-saving therapies. Thus, future approaches aimed at improving harmonization/interaction between HTA bodies and regulatory agencies should build on these existing models/mechanisms while examining their long-term impacts. Several barriers including legal, organizational, and resource-related factors were also identified, and these need to be addressed to achieve greater alignment in the current regulatory and reimbursement landscape.

## Author Contributions

RO-A and MLDB contributed to study conception, analysis, and manuscript preparation. CEH contributed to manuscript preparation and revision for intellectual content. All authors read and approved the final manuscript version prior to submission.

## Conflict of Interest

RO-A, CEH, and MLDB are employees of the Copenhagen Centre for Regulatory Sciences (CORS). CORS is a cross-faculty university anchored institution involving various public (Danish Medicines Agency, Copenhagen University) and private stakeholders (Novo Nordisk, Lundbeck, Ferring Pharmaceuticals, LEO Pharma) as well as patient organizations (Rare Diseases Denmark). The center is purely devoted to the scientific aspects of the regulatory field and with a patient-oriented focus and the research is not company-specific product or directly company related. In the last 5 years, CORS has received funding from Novo Nordisk, Lundbeck, Ferring Pharmaceuticals, and LEO pharma for projects not related to this study.
