# Supplementary material for: Improving Interactions Between Health Technology Assessment Bodies and Regulatory Agencies: A Systematic Review and Cross-Sectional Survey on Processes, Progress, Outcomes, and Challenges
Source: Front Med (Lausanne). 2020 Oct 16;7:582634. doi: 10.3389/fmed.2020.582634 (PMC7596325; doi:10.3389/fmed.2020.582634)
Supplement: Supplementary file 1 [file Data_Sheet_1.docx]

**Supplementary materials**

**Table S1:** Search strategy for decision making processes for HTA and regulatory agencies

| 1. Decision mak*.mp. OR decision-mak*.mp. OR decision process*.mp. OR decision technique*.mp. OR (decision-making).m_titl OR (decision-making process).m_titl. OR (decision making).m_titl. OR decision-making approach OR exp decision making/ OR (decision techniques).m_titl. OR (decision-making strategies).m_titl. 2. (Health technology assessment).mp. OR exp technology assessment, biomedical/ OR (health technology assessment).m_titl. OR HTA.mp. OR HTA.m_titl. OR cost-effectiveness analysis.mp. or exp Cost-Benefit Analysis/ OR (cost-effectiveness analysis).m_titl. OR (cost-benefit analysis).m_titl. OR cost-benefit analys* OR economic evaluation*.mp. OR (economic evaluation).m_titl. OR cost-utility analys*.mp. OR (cost-utility analysis).m_titl. OR health economic evaluation*.mp. OR (health economic evaluation).m_titl. OR cost-benefit evaluation*.mp. OR (cost-benefit evaluation).m_titl OR technology assessment*.mp. OR (technology assessment).m_titl. OR economic analys*.mp. OR (economic analysis).m_titl. 3. Exp drug approval/ OR (drug approval).mp. OR (drug approval).m_titl. OR (pharmaceutical approval).mp. OR (pharmaceutical approval).m_titl. OR (drug regulation).mp. OR (drug regulation).m_titl. OR (pharmaceutical regulation).mp. OR (pharmaceutical regulation).m_titl. OR exp legislation, Drug/ OR (drug legislation).mp. OR (drug legislation).m_titl. OR (pharmaceutical legislation).mp. OR (pharmaceutical legislation).m_titl. OR [government regulation*](about:blank) OR (pharmaceutical administration).mp. OR (pharmaceutical administration).m_titl. OR exp [government regulation/](about:blank) OR (government regulation).m_titl. OR (government regulation).mp. OR exp drug industry/ OR (drug industry).mp. OR (drug industry).m_titl. OR (drug industry legislation).mp. OR (drug industry legislation).m_titl. OR (drug standards).mp. OR (drug standards).m_titl. OR (pharmaceutical standards).mp. OR (pharmaceutical standards).m_titl. OR (pharmaceutical approval).mp. OR (pharmaceutical approval).m_titl. OR ([Drug Industry jurisprudence](about:blank)).mp. OR (Drug Industry jurisprudence).m_titl. OR exp "United States Food and Drug Administration"/ OR FDA.m_titl. OR (Food and drug administration).m_titl. Or EMA OR (European medicines agency).m_titl. 4. 2 OR 3 5. 1 AND 4 6. Limit 5 to (English language and humans) 7. Limit 7 to English language |
| --- |

**Table S2:** Search strategy for synergies between HTA and regulatory agencies

| 1. (Health technology assessment).mp. OR exp technology assessment, biomedical/ OR (health technology assessment).m_titl. OR HTA.mp. OR HTA.m_titl. OR cost-effectiveness analysis.mp. or exp Cost-Benefit Analysis/ OR (cost-effectiveness analysis).m_titl. OR (cost-benefit analysis).m_titl. OR cost-benefit analys* OR economic evaluation*.mp. OR (economic evaluation).m_titl. OR cost-utility analys*.mp. OR (cost-utility analysis).m_titl. OR health economic evaluation*.mp. OR (health economic evaluation).m_titl. OR cost-benefit evaluation*.mp. OR (cost-benefit evaluation).m_titl OR technology assessment*.mp. OR (technology assessment).m_titl. OR economic analys*.mp. OR (economic analysis).m_titl. 2. Exp drug approval/ OR (drug approval).mp. OR (drug approval).m_titl. OR (pharmaceutical approval).mp. OR (pharmaceutical approval).m_titl. OR (drug regulation).mp. OR (drug regulation).m_titl. OR (pharmaceutical regulation).mp. OR (pharmaceutical regulation).m_titl. OR exp legislation, Drug/ OR (drug legislation).mp. OR (drug legislation).m_titl. OR (pharmaceutical legislation).mp. OR (pharmaceutical legislation).m_titl. OR [government regulation*](about:blank) OR (pharmaceutical administration).mp. OR (pharmaceutical administration).m_titl. OR exp [government regulation/](about:blank) OR (government regulation).m_titl. OR (government regulation).mp. OR exp drug industry/ OR (drug industry).mp. OR (drug industry).m_titl. OR (drug industry legislation).mp. OR (drug industry legislation).m_titl. OR (drug standards).mp. OR (drug standards).m_titl. OR (pharmaceutical standards).mp. OR (pharmaceutical standards).m_titl. OR (pharmaceutical approval).mp. OR (pharmaceutical approval).m_titl. OR ([Drug Industry jurisprudence](about:blank)).mp. OR (Drug Industry jurisprudence).m_titl. OR exp "United States Food and Drug Administration"/ OR FDA.m_titl. OR (Food and drug administration).m_titl. Or EMA OR (European medicines agency).m_titl. 3. 1 AND 2 4. Synerg*.mp. OR synerg.mp. OR synergy.m_titl. OR synergies.m_titl. OR align*.mp. OR alignment.m_titl. OR alignments.m_titl. OR collaborat*.mp. OR collaborate.m_titl. OR collaboration.m_titl. OR engage*.mp. OR engage.m_titl. OR engagement.m_titl. OR agree* OR agreement.m_titl. OR disagree*.mp. OR disagreement.m_titl. OR alliance* OR alliance.m_titl. OR partner*.mp. OR partnership.m_titl. OR harmon*.mp. OR hamony.m_titl. OR hamonisation.m_titl. OR harmonization.m_titl. OR confluence*.mp. OR confluence.m_titl. OR collision*.mp. OR collision.m_titl. OR standard*.mp. OR standardisation.m_titl. OR standardization.m_titl. OR cooperate*.mp. OR cooperation.m_titl. OR interact*.mp. OR interaction.m_titl. OR parallel scientific advice.mp. OR (parallel scientific advice).m_titl. OR (Parallel Regulatory-HTA Scientific Advice).m_titl. OR scientific advice.mp. OR (scientific advice).m_titl. OR parallel consultation*.mp. OR (parallel consultation).m_titl. 5. 3 AND 4   6 Limit 5 to (English language and humans) |
| --- |

**Table S3:** List of HTA bodies and websites

| **Country** | **HTA body (formal)** | **Other HTA bodies** | **Website** |
| --- | --- | --- | --- |
| Europe | n/a | EUnetHTA: European Network for Health Technology Assessment  Health Evidence Network (HEN) EuroScan - The European Information Network on New and Changing Health Technologies EURONHEED Database – Health economics Central and Eastern European Society for TA in Health Care (CEESTAHC) | http://www.eunethta.net/  http://www.euro.who.int/hen  http://www.euroscan.bham.ac.uk/  http://infodoc.inserm.fr/euronheed/Publication.nsf |
| International | n/a | INAHTA - The International Network of Agencies for Health Technology Assessment Health Technology Assessment International (HTAi) ISPOR global health care system roadmap WHO Essential Health Technologies (EHT) |  |
| Albania | n/a | MoH, Dept of General Policy Planning and Health Pharmaceutical Directorate | n/a |
| Armenia | n/a | n/a | n/a |
| Australia | Pharmaceutical Benefit Advisory Committee (PBAC)/ Medical Services Advisory Committee (MSAC) | National Health and Medical Research Council Australia  Victorian Policy Advisory Committee on Clinical Practice and Technology Centre for Health Program Evaluation (Monash University) The Centre for Clinical Effectiveness (CCE) Adelaide Health Technology Assessment (Committee) (AHTA) Australian Safety and Efficacy Register of New Interventional Procedures – Surgical (ASERNIP-S) Centre for Health Economics Research and Evaluation (CHERE) Australia and New Zeland Horizon Scanning Network (ANZHSN) Social and Public Health Economics Research Group (SPHERE) University for Health Sciences, Medical Informatics and Technology | http://www.msac.gov.au/  http://www.nhmrc.gov.au/  http://www.health.vic.gov.au/newtech/committee.htm  http://www.buseco.monash.edu.au/centres/che/ http://www.mihsr.monash.org/cce/ http://www.adelaide.edu.au/ahta/ http://www.surgeons.org/asernip-s/ http://www.chere.uts.edu.au/ http://www.horizonscanning.gov.au/ http://sphere.curtin.edu.au/ |
| Austria | Austrian Federal Institute for Health Care (ÖBIG) / Austrian Institute of Technology Assessment | Gesundheit Osterreich GmBH, Austrian Health Institute (GoG) Ludwig Boltzmann Institute of Health Technology Assessment (LBI for HTA) Institute for Pharmaeconomic Research (IPF)  University for Health Sciences, Medical computer science and engineering Institute of Public Health, Medical Decision Making and HTA  Department for Evidence-Based Medicine and Clinical Epidemiology , Danube University Krems | http://www.oeaw.ac.at/ita/welcome.htm  http://hta.lbg.ac.at/de/index.php  http://www.ipf-ac.at  http://www.umit.at/page.cfm?vpath=departments/public_health |
| Belgium | Belgian Health Care Knowledge Centre (KCE)  National institute for Health and Disability Insurance | Centre for Health Services Research Katholieke Univesiteit Leuven Research and Development | [http://www.kce.fgov.be/Index.aspx?SGREF=3232](about:blank)  [https://www.inami.fgov.be/fr/Pages/default.aspx](about:blank) |
| Bosnia and Herzegovina | HTA board by MoH (recently established) | n/a | http://www.alims.gov.ba/index.html www.farmakoekonomika.ba |
| Bulgaria | n/a | Committee for the Positive Drug List National Centre of Public Health Protection (NCPHP) |  |
| Canada | Canadian Agency for Drugs and Technologies in Health (CADTH) | Institut National d'Excellence en Sante et Services Sociaux (INESS) Alberta Heritage Foundation for Medical Research (AHFMR) British Columbia Centre for Health Services and Policy Research (CHSPR) Institute for Clinical Evaluative Sciences (ICES) Institute of Health Economics (IHE) Centre for Health Economics and Policy Research (CHEPA) Centre for Health Services and Policy Research (CHSPR) Calgary Institute for Population and Public Health (CIPPH) Toronto Health Economics and Technology Assessment (THETA)  Centre for Health Evaluation and Outcome Sciences (CHEOS) Centre Hospitalier de l'Université de Montréal (CHUM) Canadian Task Force on Preventive Health Care (CTFPHC) Health Quality Council (HQC) Technology Assessment Unit of the McGill University Health Centre (MUHC) Medical Advisory Secretariat, Ontario Ministry of Health and Long-Term Care (MAS) Ontario Health Technology Advisory Committee (OHTAC) Programs for Assessment of Technology in Health, McMaster University (PATH) | http://www.inesss.qc.ca/ http://www.ahfmr.ab.ca/ http://www.chspr.ubc.ca/ http://path-hta.ca/report.htm  http://www.cadth.ca/index.php/en/home http://www.ices.on.ca/webpage.cfm http://www.ihe.ca/  http://www.chepa.org/Home.aspx  http://www.chspr.ubc.ca/ http://www.ucalgary.ca/cipph/ http://theta.utoronto.ca/ http://www.cheos.ubc.ca/ http://www.chumtl.qc.ca/notre-equipe/directions/detmis.fr.html  http://www.ctfphc.org/  http://healthcouncilcanada.ca/en/index.php http://www.mcgill.ca/tau/  http://www.health.gov.on.ca/english/providers/program/mas/mas_mn.html |
| China (Hong Kong) | n/a | School of Pharmacy, Faculty of Medicine, The Chinese University of Hong Kong |  |
| Croatia | Agency for Quality and Accreditation in Health Care, Croatia Department for Development, Research and HTA | n/a | http://www.aaz.hr/main.php?ID=4 |
| Cyprus | n/a | n/a | http://www.moh.gov.cy/moh/moh.nsf/dmlhealth_en/dmlhealth_en?OpenDocument |
| Czech Republic | National Reference Centre/Drug Categorization Committee | n/a | http://www.nrc.cz/ |
| Democratic People's Republic of Korea | n/a | n/a | n/a |
| Denmark | Danish Centre for Evaluation and HTA (DACEHTA) | Centre for Applied Health Services Research and Technology Assessment (CAST) Danish Institute for Health Services Research and Development (DSI)  Odense Universitetshospital, Department of Quality and Research, Health Technology Assessment Centre for Applied Health Services Research and Technology Assessment (CAST) Centre for Public Health, MTV og Sundhedstjeneste- forskning  National Board of Health (NBoH) Institute for Rational Pharmacotherapy (IRF) | http://www.sst.dk/Planlaegning_og_behandling/Medicinsk_teknologivurdering.aspx?lang=en http://www.dsi.dk/  http://www.ouh.dk/wm134768 http://www.sdu.dk/om_sdu/institutter_centre/cast http://www.centerforfolkesundhed.dk/om+centret/in+english |
| Estonia | n/a | Pharmaceutical Committee and State Agency of Medicines  Estonian Health Insurance Fund (EHIF) University of Tartu-Department of Public Health (UTA) | http://www.haigekassa.ee/kindlustatule/soodusravimid/hindamine/ |
| Finland | Finnish Office for Health Technology Assessment (FinOHTA) | n/a | http://finohta.stakes.fi/EN/index.htm |
| France | Agence Nationale d’Accreditation et d’Evaluation en Santé et Haute Autorité de Santé (ANAES and HAS) | Agence Nationale pour le Developpement de l’Evaluation Medicale (ANDEM) Comité d'Evaluation et de Diffusion des Innovations Technologiques Assistance Publique Hôpitaux de Paris, France (CEDIT) Agence Francaise de Securite Sanitaire des Produits de Sante (AFSSAPS) REES - Reseau d'Evaluation en Economie de la Sante | http://www.has-sante.fr/portail/display.jsp?id=j_5 http://upml.fr/andem/andem.htm  http://cedit.aphp.fr/  http://www.rees-france.com/ |
| Germany | The Institute for Quality and Efficiency in Health Care (IQWiG) | German Institute for Medical Documentation and Information (DIMDI)  Hannover Medical School, Medizinische Hochschule Hannover (MHH) Institute for Public Health and Nursing Research, IPP Bremen Department of Health Services Research, Faculty for Human Sciences and Health Sciences, The University of Bremen German Health Care System and the Federal Joint Committee (G-ba) | http://www.dimdi.de/static/en/index.html http://www.iqwig.de/ http://www.mh-hannover.de/  http://www.g-ba.de |
| Greece | National Organisation for Medicines (EOF) | Center for Health Services Management and Evaluation, University of Athens National School of Public Health (NSPH/ESDY) | http://www.eof.gr/ http://chesme.nurs.uoa.gr/eng/  http://www.nsph.gr/default.aspx?page=home |
| Hungary | Health Economics and Technology Assessment Research Centre (HunHTA) | Technology Appraisal Committee (TAC) Office of HTA (OHTA) Hungarian Coordination Office for HTA (HCOHTA) National Institute for Quality and Organizational Development in Healthcare and Medicines (GYEMSZI) | http://hecon.uni-corvinus.hu/  http://www.medinfo.hu/new3/technologia_en/technologia_en.php http://gyemszi.hu/ |
| Iceland | n/a | n/a | http://www.velferdarraduneyti.is/raduneyti/um-raduneytid/ |
| Ireland | Health Information and Quality Authority (HIQA) | Health Economics Association of Ireland (HEAI) Medicines Board National Centre for Pharmacoeconomics (NCPE) | http://www.dohc.ie/ http://www.hiqa.ie/publications.asp http://www.ncpe.ie/index.php |
| Israel | Israel Center for Technology Assessment in Health Care (ICTAHC) | n/a | http://www.gertnerinst.org.il/e/health_policy_e/technology/ http://www.health.gov.il/english/ |
| Italy | L'Agenzia nazionale per I servizi sanitari regionali, the Agency for Regional Healthcare (Age.Na.S) | HTA Unit in A. Gemelli Teaching Hospital (UVT) OSMED Coordination Office and the activities of HTA Laziosanita, Agenzia di Sanita Pubblica, Regione Lazio Reglom-DGSAN, Regione Lombardia Direzione Generale Sanita Regione Emilia Romagna, Agenzia Sanitaria e Sociale Regione Emilia Romagna (ASSR) Regione Veneto, Direzione Piani e Programmi Socio Sanitari | http://www.assr.it/ http://www.policlinicogemelli.it/area/?s=206 http://asr.regione.emilia-romagna.it/ |
| Japan | Institute of Healthcare Technology Assessment, Shomachi/ Department of Technology Assessment and Biostatistics | Technology Assessment & Decision Science Unit College of Life Sciences, Ritsumeikan University Department of Health Economics and Epidemiology Research, University of Tokyo | http://www.niph.go.jp/English/research/01techno/index.html |
| Latvia | Health Statistics and Medical Technology State Agency (VSMTA) | State Medicines Pricing and Reimbursement Agency Centre for Health Economics (VEC) | http://www.vm.gov.lv/index.php?setlang=en http://vec.gov.lv/en |
| Leichtenstein | n/a | n/a | http://www.llv.li/amtsstellen/llv-ag-home.htm |
| Lithuania | State Health Care Accreditation Agency under the MoH (VASPVT) | Pharmaceutical Reimbursement Committee | [http://www.vaspvt.gov.lt/en](about:blank) |
| Luxembourg | n/a | Cellule d'Expertise Medicale (CEM) Centre de Recherche Public de la Sante (CRP-Sante) | http://www.ms.public.lu/fr/index.html |
| Malta | Pharmaceutical HTA Unit | Ministry for Social Policy/Strategy and Sustainability Division (SSD/MSOC) | [https://ehealth.gov.mt/HealthPortal/strategy_policy/pharm_pol_mon/pharm_hta_unit.aspx](about:blank) |
| Monaco | n/a | n/a | http://www.gouv.mc/devwww/wwwnew.nsf/ |
| Netherlands | National Health Care Institute |  | [https://english.zorginstituutnederland.nl/about-us](about:blank) |
| New Zealand | Pharmacology and Therapeutics Advisory Committee (PTAC)/ New Zealand Health Technology Assessment (NZHTA) | Core Services Committee Health Services Assessment Collaboration (HSAC) | http://www.healthsac.net/index.htm http://nzhta.chmeds.ac.nz/ |
| Norway | Norwegian Knowledge Centre for the Health Services (NOKC) | Health Services Research Unit Institute of Community Medicine SINTEF-UNIMED | http://www.kunnskapssenteret.no/ |
| Poland | Agencja Oceny Technologii Medycznych, Agency for Health Technology Assessment in Poland (AHTAPol) | Association for Quality Promotion (TPJ) Centre for Farmacoeconomics Polish Society for Pharmacoeconomics Central and Eastern European Society for Technology Assessment in Health Care (CEESTAHC) | http://www.tpj.pl/hta.php http://www.farmakoekonomika.edu.pl/ http://www.aotm.gov.pl/ http://www.farmakoekonomika.pl/ |
| Portugal | National Institute of Pharmacy and Medicines (INFARMED) | n/a | http://www.infarmed.pt/portal/page/portal/INFARMED/ENGLISH |
| Republic of Korea (South) | Health Insurance Review and Assessment Agency (HIRA) | National Evidence-based Healthcare Collaborating Agency (NECA) Korea Association of HTA (KAHTA) Committee for New Health Technology Assessment (CNHTA) | http://www.hira.or.kr/cms/rb/rbb_english/index.html?pgmid=HIRAB960000000000 www.neca.re.kr  http://www.kahta.or.kr/ |
| Romania | n/a | National School of Public Health, Management and Professional Development (SNSPMS) |  |
| Serbia | n/a | Medicines and Medical Devices Agency of Serbia (ALIMS) Quality Unit, Ministry of Health Serbia | http://www.alims.gov.rs/ |
| Singapore | Research and TA Department | n/a | http://www.singhealth.com.sg/Research/HealthServicesResearch/OurServices/HealthTechnologyAssessment/Pages/Home.aspx  http://www.moh.gov.sg/mohcorp/publications.aspx?id=92 |
| Slovakia (Slovak Republic) | Slovak Agency for HTA (SLOVATHA) | n/a | http://www.health.gov.sk/ http://www.farmako-ekonomika.sk/main.php?skok=60&idnew=353 |
| Slovenia | Agency for medicinal products and medical devices of the republic of slovenia (JAZMP) | Health Insurance Institute Institute of Public Health of the Republic of Slovenia (NIPH-RS) Institute for Economic Research (IER), Ministry of Health, Slovenia | http://www.jazmp.si/index.php?id=1 |
| Spain | Agencia de Evaluación de Tecnologías Sanitarias (Instituto de Salud Carlos III) (AETS) | Catalan Agency for Health Information, Assessment, and Quality (CAHIAQ formely CAHTA)  Agencia de Evaluación de Tecnologías Sanitarias de Andalucía (AETSA) Basque Office for Health Technology Assessment (OSTEBA) Agencia de Evaluación de Tecnologías Sanitarias de Galicia (AVALIA-T) Agencia de Evaluación de Tecnologías Sanitarias de Canarias Agencia de Evaluación de Tecnologías Lain Entralgo, also host of Unidad de Evaluacion de Tecnologias Sanitarias (UETS) Ministry of Health and Social Policy | http://www.isciii.es/htdocs/index.jsp http://www.gencat.net/salut/depsan/units/aatrm/html/en/dir394/index.html http://www.juntadeandalucia.es/salud/orgdep/aetsa/  http://www.osasun.ejgv.euskadi.net/r52-2536/es/ http://www.sergas.es/MostrarContidos_Portais.aspx?IdPaxina=60538&Idioma=es http://www.gobiernodecanarias.org/sanidad/sescs/ http://www.madrid.org/cs/Satellite?c=CM_Actuaciones_FA&cid=1142402651366&idConsejeria=1109266187266&idListConsj=1109265444710&idOrganismo=1109266228196&language=es&pagename=ComunidadMadrid%2FEstructura&pid=1109265444699&sm=1109266100977 |
| Sweden | Swedish Council on Technology Assessment in Health Care (SBU)/Dental and Pharmaceutical Benefits Agency (TLV) | Center for Medical Technology Assessment, Linköping University (CMT) Center for evidence-based medicine and health technology assessment in Örebro County Council (OCC) HTA Center Western Götlandsregionen (CWG) The Swedish Institute for Health Economics (IHE) Centre for evidence-based medicine and evaluation of medical training in the Örebro County Council (CAMTO) | http://www.sbu.se/en/ http://www.cmt.liu.se/  http://www.orebroll.se/uso/page____2834.aspx  http://www.sahlgrenska.se/sv/SU/Forskning/HTA-centrum/Hogerkolumn/Genomforda-mini-HTA/  http://www.ihe.se/ http://www.orebroll.se/sv/uso/Forskning/Forskningsenheter/CAMTO/ |
| Switzerland | Swiss Centre for Technology Assessment (TA-SWISS)/ Medical Technology Unit - Swiss Federal Office of Public Health (MTU-SFOPH) | Swiss Network for Health Technology Assessment (SNHTA) Federal Office of Public Health Medical Advisors Section | http://www.ta-swiss.ch/en/ http://www.snhta.ch/home/portal.php http://www.bag.admin.ch/ |
| Ukraine | n/a | n/a |  |
| United Kingdom (England) | National Institute for Health and Care Excellence (NICE) | n/a | http://www.nice.org.uk/ |
| United Kingdom (Scotland) | Scottish Medicines Consortium (SMC) | n/a | http://www.sign.ac.uk/ |
| United Kingdom (Wales) | All Wales Medicines Strategy Group (AWMSG) | n/a | https://awmsg.nhs.wales/ |
| United States of America | Agency for Healthcare Research and Quality (AHRQ) Centre for Medical Technology Policy (CMTP) | American health care provider (AETNA) Academy of Managed Care Pharmacy (AMCP)  Foundation of Managed Care Pharmacy (FMCP)  Health Services/Technology Assessment Text, NLM (HSTAT database) National Information Center on Health Services Research and Health Care Technology (NICHSR) Centre for Drug Evaluation and Research (CDER) *drugs only ECRI (formerly Emergency Care Research Institute)* free access through MDT elibrary Health Technology Advisory Committee, Minnesota Department of Health (HTAC) HAYES Inc – an independent health technology assessment organization University Health System Consortium (UHC) The Institute for Clinical Systems Improvement (ICSI ) Medical Technology and Practice Patterns Institute (MTPPI) National Guidelines Clearinghouse (NGC) VA Technology Assessment Program (VATAP) | http://www.aetna.com/cpb/cpb_menu.html  http://www.ncbi.nlm.nih.gov/books/bv.fcgi?rid=hstat  http://www.nlm.nih.gov/nichsr/nichsr.html  http://www.fda.gov/AboutFDA/CentersOffices/CDER/default.htm http://www.ecri.org/Pages/default.aspx http://www.health.state.mn.us/htac/  http://www.hayesinc.com/  http://www.uhc.edu/  http://www.icsi.org/index.aspx  http://www.mtppi.org/frameset.asp?Pg=/&MI=1  http://www.guideline.gov/ http://www.va.gov/vatap |

**Table S4:** List of regulatory agencies

| **Country** | **Agencies** | **Websites** |
| --- | --- | --- |
| Multi-national | World Health Organization  International organization for standardization  International conference on harmonization | [https://www.who.int/](about:blank)  [https://www.iso.org/home.html](about:blank)  [https://www.ich.org/home.html](about:blank) |
| Americas | Pan American Health Organization | [https://www.paho.org/hq/index.php?lang=en](about:blank) |
| Europe | European medicines Agency | [https://www.ema.europa.eu/en](about:blank) |
| Australia | Therapeutics goods administration | [https://www.tga.gov.au/](about:blank) |
| China | State Food and Drug administration | [http://sfda.com/](about:blank) |
| Denmark | Danish Medicines Agency | [https://laegemiddelstyrelsen.dk/en/Borgere/](about:blank) |
| Estonia | State Agency of Medicines | [https://ravimiamet.ee/en](about:blank) |
| Finland | National agency for medicines | [https://www.fimea.fi/web/en](about:blank) |
| France | National agency for the safety of medicine and health products | [http://www.ansm.sante.fr/](about:blank) |
| Germany | Federal Institute for Drugs and Medical Devices | [http://www.bfarm.de/](about:blank) |
| Greece | National organization for Medicines | [http://www.eof.gr/](about:blank) |
| Hong Kong | Department of Health, Pharmaceutical sciences | [https://www.dh.gov.hk/](about:blank) |
| Hungary | National Institute of pharmacy and nutrition | [http://www.ogyei.gov.hu/](about:blank) |
| Iceland | Icelandic medicines agency | [http://www.ima.is/](about:blank) |
| Ireland | Health Products Regulatory Authority | [https://www.hpra.ie/](about:blank) |
| Latvia | State agency of medicines | [http://www.zva.gov.lv/](about:blank) |
| Liechtenstein | Office of health/ Department of pharmaceuticals | [https://www.llv.li/](about:blank) |
| Lithuania | State medicine control agency | [http://www.vvkt.lt/](about:blank) |
| Luxembourg | Ministry of Health | [http://www.ms.etat.lu/](about:blank) |
| Malta | Medicines authority | [http://www.ms.etat.lu/](about:blank) |
| Israel | Ministry of Health | [https://www.health.gov.il/English/Pages/HomePage.aspx](about:blank) |
| Italy | Italian Medicines Agency | [https://www.aifa.gov.it/](about:blank) |
| Japan | Pharmaceutical and Medical devices agency | [http://www.pmda.go.jp/english/index.html](about:blank) |
| Netherlands | Medicines Evaluation Board  Healthcare inspectorate | [https://english.cbg-meb.nl/](about:blank)  [http://www.igz.nl/](about:blank) |
| New Zealand | Medicines and Medical devices safety authority | [https://www.medsafe.govt.nz/](about:blank) |
| Norway | Ministry of Health and Care Services | [https://www.regjeringen.no/en/dep/hod/id421/](about:blank) |
| Poland | Office for registration of medicinal products, medical devices and biocidal products  Chief pharmaceutical inspectorate | [http://www.urpl.gov.pl/](about:blank)  [http://www.gif.gov.pl/](about:blank) |
| Portugal | National authority of medicines and health products | [http://www.infarmed.pt/](about:blank) |
| Romania | National authority of medicines and medical devices | [http://www.anm.ro/](about:blank) |
| Slovakia | State institute for drug control | [http://www.sukl.sk/](about:blank) |
| Slovenia | Agency for Medicinal Products and Medical Devices of the Republic of Slovenia | [http://www.jazmp.si/](about:blank) |
| Russia | Public health institute | [https://public-health.ru/en](about:blank) |
| Serbia | Ministry of Health | [https://www.zdravlje.gov.rs/](about:blank) |
| South Korea | Ministry of food and drug safety | [https://www.mfds.go.kr/eng/index.do](about:blank) |
| Spain | Spain agency for medicines and health products | [http://www.aemps.gob.es/](about:blank) |
| Sweden | Medical Products Agency | [http://www.lakemedelsverket.se/](about:blank) |
| Switzerland | Swiss Agency for therapeutic products | [https://www.swissmedic.ch/swissmedic/en/home/about-us/swissmedic--swiss-agency-for-therapeutic-products.html](about:blank) |
| Turkey | Ministry of Health | [https://www.saglik.gov.tr/?_Dil=2](about:blank) |
| Ukraine | Ministry of Health | [https://en.moz.gov.ua/](about:blank) |
| UK | Medicines and healthcare products regulatory agency | [https://www.gov.uk/government/organisations/medicines-and-healthcare-products-regulatory-agency](about:blank) |
| USA | Food and drug administration | [https://www.fda.gov/](about:blank) |

**Table S5:** Examples of large EU funded projects that will be reviewed for relevant information

| **Project** | **Link** |
| --- | --- |
| *EUnetHTA Joint action 1,2, 3*: Collaboration of all HTA bodies in Europe | [https://www.eunethta.eu/category/activities/eunethta-joint-action-3-2016-2020/](about:blank) ;  [https://ec.europa.eu/health/technology_assessment/joint_actions_en](about:blank)  [https://www.eunethta.eu/ja1-archive/](about:blank)  [https://www.eunethta.eu/ja2-archive/](about:blank) |
| *IMI Project PREFER – patient preferences in benefit-risk assessments:* Studies how to determine and include patient-preference in decision making during the drug life cycle. | [https://www.imi-prefer.eu/](about:blank)  [https://www.imi.europa.eu/](about:blank) |
| *IMI BigDATA HARMONY:* Public-private partnership aiming to improve the outcomes of patients with hematological malignancies via the sharing of big data among all stakeholders. | [https://www.imi.europa.eu/news-events/events/big-data-health-imis-harmony-project](about:blank)  [https://www.harmony-alliance.eu/](about:blank) |
| *H2020 IMPACT HTA (improved methods and actionable tools for enhancing HTA):* proposes new and improved methods, tools and guidance for decision-making across in the context of HTA and health system performance measures. | [https://www.impact-hta.eu/](about:blank) |
| *H2020 COMED:* Pushing the boundaries of cost and outcome analysis of medical technology | [http://www.comedh2020.eu/wps/wcm/connect/Site/COMED/Home](about:blank)  [https://cordis.europa.eu/project/rcn/213046/factsheet/en](about:blank) |
| *IMI GetReal Initiative (Real life data in drug development):* Aims to show robust new methods for collecting and synthesizing real world data that can contribute to pharmaceutical R&D and healthcare decision making. | [https://www.imi-getreal.eu/](about:blank)  [https://www.imi.europa.eu/projects-results/project-factsheets/getreal-initiative](about:blank) |

**Table S6:** Short online survey sent to HTA bodies*

| **Q1:** What is your name?  **Q2:** Is there a formal link between your agency and  HTA bodies? Yes/No  **Q3a:** Have there been any collaborative initiatives between your organisation and HTA bodies? Yes/No  **Q3b:** If yes, what are the key areas of collaboration?  **Q4:** What have been the key outcomes, challenges and learning points from synergy initiatives with HTA bodies?  **Q5:** Is there anything else you want to report regarding collaborative efforts with HTA bodies?  **Q6a:** Do you think some of your operations can be jointly performed with HTA agencies?  **Q6b:** If yes to the above question, which ones? |
| --- |

*The wording was reversed for regulatory agencies

**Table S7:** Overview of key publications informing key themes for the report

| Reference | Type of document | Key highlight |
| --- | --- | --- |
| Eichler et al^2^ | Journal article | Discusses the methodology of relative efficacy and effectiveness |
| Katz^4^ | Journal article | Good overview of evidentiary standards for drug approval |
| Liberti et al^19^ | Journal article | Commentary on cooperation between regulatory agencies, sponsors and HTA bodies |
| Tsoi et al^3^ | Journal article | In-depth review of harmonization of reimbursement and regulatory processes |
| Tsoi et al^100^ | Journal article | Presents results of interview among Canadian HTA assessors and regulators concerning synergies |
| Henshall et al^20^ | Journal article | Summaries discussion in an HTA assessors of their views about synergy with regulatory |
| Fronsdal et al^51^ | Journal article | Good overview of initiatives of HTA regulatory synergy |
| Wang et al^25^ | Journal article | Explores potential areas of synergy via questionnaire |
| Hutton et al^22^ | Journal article | Discussions on harmonization of evidence requirements for HTA |
| Berntgen et al^42^ | Journal article | A report on the contribution of regulatory reports to HTA |
| Tafuri et al^57^ | Journal article | Examines the impact of HTA-regulatory parallel advice on clinical development programs |
| Tafuri et al^45^ | Journal article | Examines the evidentiary alignment of HTA and regulatory during early dialogue |
| Khan et al^53^ | Journal article | Commentary on industry perspectives of parallel consultation |
| Dekker et al^46^ | Journal article | Assesses the evidentiary alignment of HTA and regulatory agencies relative to dementia medicines |
| Wonder et al^60^ | Journal article | Describes pilot scientific program in Australia |
| Backhouse et al^50^ | Journal article | Describes HTA early dialogue |
| Ridge et al^72^ | Journal article | Describes an experience with the US FDA-CMS parallel submission program |
| Eichler et al^80^ | Journal article | Discusses concept of adaptive licensing |
| Eichler et al^82^ | Journal article | Discusses concept of adaptive pathways to medicine access |
| Bouvy et al^78^ | Journal article | Discusses HTA within adaptive pathways |
| Pearce et al^64^ | Journal article | Examines timelines of PBAC decisions after TGA approval in Australia |
| McAuslane et al^61^ | Journal article | Presents findings from a workshop discussion on HTA and regulatory harmonization |
| Bramley et al^56^ | ISPOR policy perspective | Discusses industry dilemma whether to seek scientific advice |
| Tschank^55^ | Master’s thesis | Examines the role of EMA-HTA engagement on drug development |
| EMA^17^ | Report | Reflection paper on synergies between HTA and regulatory agencies |
| EMA^10^ | Report | Road map to 2015, which discusses EMA’s contribution to science, Medicine and Health |
| Panteli et al^5^ | Policy brief | Discusses important concepts around patient access |
| GSK^66^ | Report | Provides extensive overview of the PBS in Australia |
| Brownsword et al^78^ | Book | Provides useful chapter on adaptive licensing |
| CRIS^65^ | Report | Reviews HTA timeliness and outcomes in Australia, Canada and Europe during 2014-2015 |
| Podemska-Mikluch^70^ | Working paper | Discusses some of the challenges of the FDA-CMUS parallel review |
| EMA^48^ | Policy Document | Provides guidance for applicants seeking scientific advice |
| National Institute for Public Health and the Environment^88^ | Report | Discusses adaptive pathways within the Dutch context |
| Liberti^69^ | Presentation | Provides overview on the impact of HTA-regulatory parallel review in Canada |
| National Institute of Medicine^27^ | Book | Discusses priorities of CER within US |
| Innovative Medicines Initiative^90^ | Website | ADAPT-SMART initiative |
| Stein et al.^83^ | Report | Provides overview of early access programs |
| EMA^23^ | Website | Provides information on parallel consultation process |
| Office of Health Economics (OHE)^21^ | Website | Provides some comments on HTA-regulatory harmonization from a workshop |
| CTMP^40^ | Website | Green Park Collaboration |
| European Commission^41^ | Report | Conclusions of 2008 Pharmaceutical forum |
| EMA^43^ | Report | EMA-EUnetHTA three-year work plan, 2013 |
| EMA^52^ | Report | Report from a workshop on EMA-HTA early dialogue |
| EMA^44^ | Report | implementation of the EMA-EUnetHTA threeyear work plan 2012-2015 |
| EMA^54^ | Report | Best practice guidance for PSA |
| NICE^58^ | Website | Scientific advice |
| Trapestry Network^59^ | Report | Consultation on multi-stakeholder involvement in drug development |
| Australian government^62^ | Website | Memorandum of understanding for parallel review |
| Department of Health, Australia^63^ | Website | TGA-PBAC parallel review process |
| CADTH^68^ | Website | CADTH common drug review |
| US Federal register^70^ | Website | FDA-CMS parallel review, 2010 |
| US Federal register^74^ | Website | FDA-CMS parallel review, 2016 |
| Netherlands Healthcare Institute^76^ | Website | Parallel Procedures MEB-ZIN |
| MaRS Discovery District^77^ | Website | MaRS project |
| EMA^87^ | Report | Final report on adaptive pathways pilot |
| EMA^93^ | Advisory document | post-authorisation procedural advice |
| US FDA^94^ | Guidance document | Post marketing studies guidance |
| EMA^98^ | Website | Patient registries |
| GET Real^99^ | Website | GET real project |
| Swedish Medical Products Agency^102^ | Website | Scientific advice MPA-TLV |

**Figure S1:** Thematic clusters of questions submitted for Parallel Scientific Advice by the EMA and HTA-bodies^55^


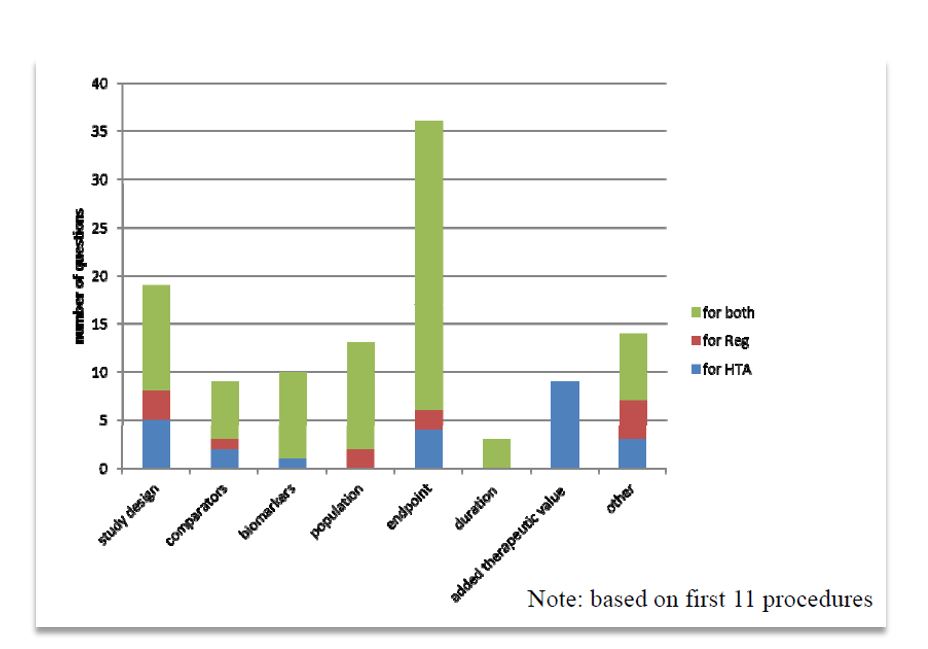


**Table S8:** Summary of proposals for adaptive licensing approach^79^

| Initiative | Details |
| --- | --- |
| Health Canada Progressive Licensing Project (2005-present) | Life cycle evidence-based strategy to drug licensing. The proposed legislation expired in 2008 but regulatory modernization efforts continue |
| US institute of Medicine, Future of drug safety (2006) | Recognizes the impossibility of understanding the effects of drugs at time of market entry and endorses (i) aggressive assessment of drug effects through life cycle; (ii) public-private funding of post market assessment (iii) overhaul of adverse events reporting (iv) investments in pharmacoepidemiology and (v) FDA to demand postmarketing reports and conduct full 5-year reviews of new molecular entities. |
| European Medicines Agency, Road Map to 2015 | Outlines staggered approval approach for situations not covered by conditional marketing authorizations, with initial focus on restricted population of good responders, followed by modification as data from real world settings become available. |
| Singapore health services authority (2011) | Expressed commitment to explore adaptive licensing for selected drugs. May enable patients and providers to have faster access to novel drugs without compromising safety through proactively accumulating clinical data via active surveillance to better understand heterogenous response to new therapies |
| UK athenaeum group (2010-present) | Multi stakeholder group that examines the limits of the current model of drug licensing and argues for flexibility in licensing, enabling in appropriate cases early access by patients while evidence continue to be collected. |

**Figure S2:** Adaptive pathways approaches^87^


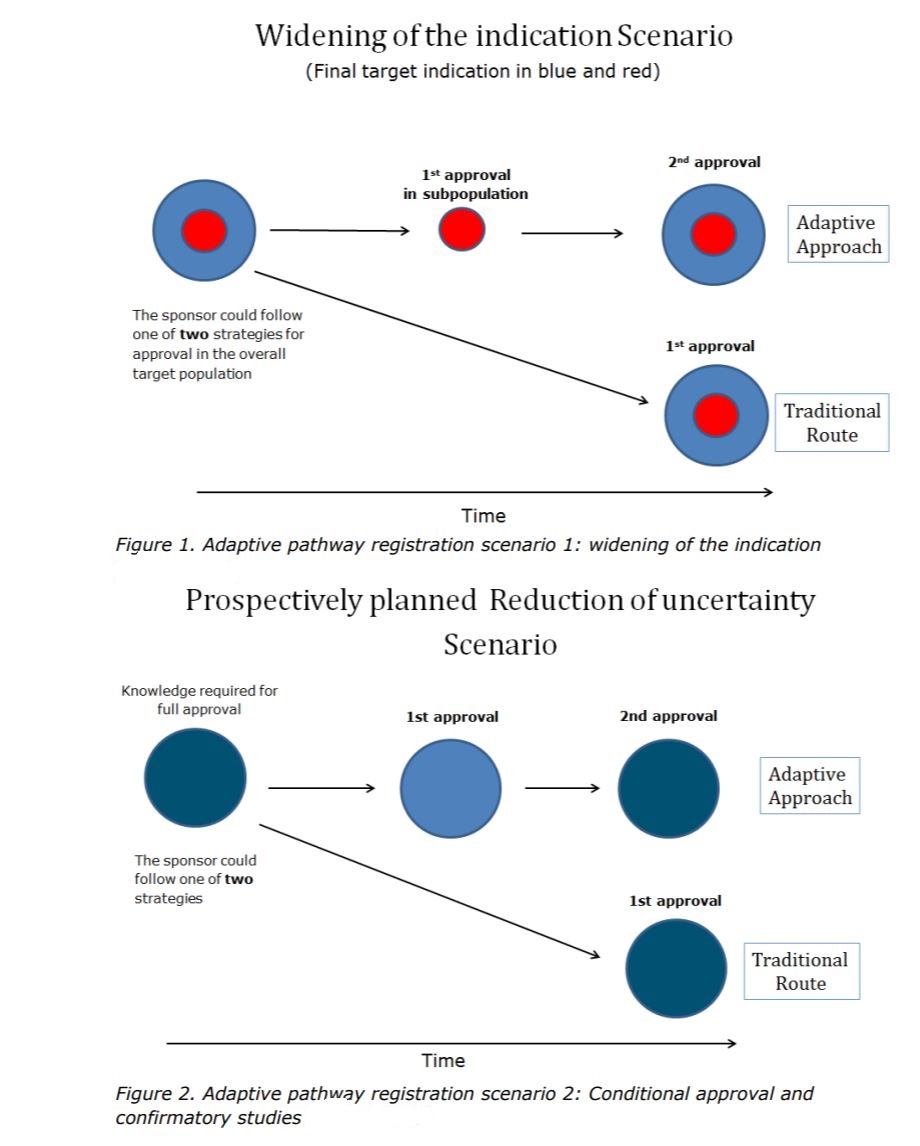


**References**

1. Clark D, Dean G, Bolton S, Beeson B. Bench to bedside: The technology adoption pathway in healthcare. *Health and Technology.* 2019.
2. Eichler HG, Bloechl-Daum B, Abadie E, Barnett D, Konig F, Pearson S. Relative efficacy of drugs: an emerging issue between regulatory agencies and third-party payers. *Nat Rev Drug Discov.* 2010;9(4):277-291.
3. Tsoi B, Masucci L, Campbell K, Drummond M, O'Reilly D, Goeree R. Harmonization of reimbursement and regulatory approval processes: a systematic review of international experiences. *Expert Rev Pharmacoecon Outcomes Res.* 2013;13(4):497-511.
4. Katz R. FDA: evidentiary standards for drug development and approval. *NeuroRx.* 2004;1(3):307-316.
5. Panteli D, Edwards S. Ensuring access to medicines: How to stimulate innovation to meet patients’ needs? 2018; [http://www.euro.who.int/__data/assets/pdf_file/0009/379701/PB29_AUSTRIA_web_13082018.pdf](about:blank). Accessed Accessed March 30, 2020.
6. Goodman C. *HTA 101: Introduction to Health Technology Assessment.* Bethesda, MD: National Library of Medicine (US); 2014.
7. Angelis A, Lange A, Kanavos P. Using health technology assessment to assess the value of new medicines: results of a systematic review and expert consultation across eight European countries. *Eur J Health Econ.* 2018;19(1):123-152.
8. Iskrov G, Dermendzhiev S, Miteva-Katrandzhieva T, Stefanov R. Health Economic Data in Reimbursement of New Medical Technologies: Importance of the Socio-Economic Burden as a Decision-Making Criterion. *Front Pharmacol.* 2016;7:252-252.
9. Panzitta M, Bruno G, Giovagnoli S, Mendicino FR, Ricci M. Drug delivery system innovation and Health Technology Assessment: Upgrading from Clinical to Technological Assessment. *Int J Pharm.* 2015;495(2):1005-1018.
10. European Medicines Agency. *Road map to 2015: The European Medicines Agency’s contribution to science, medicines and health.* London: EMA; 2011.
11. Mossialos E, Mrazek M, Walley T. *Regulating pharmaceuticals in Europe: striving for efficiency, equity and quality. .* Copenhagen: European Observatory on Health Systems and Polices; 2004.
12. Grignolo A, Siu A. Improving Drug Development and Patient Access With the Right People, Processes, and Culture: What Needs to Happen Right Now to Bring Better Medicines to the Patients Who Need Them. *Ther Innov Regul Sci.* 2019;53(3):398-402.
13. IQVIA Institute for Human Data Science. Global Oncology Trends 2018: Innovation, Expansion and Disruption. 2018; [www.iqvia.com/institute/reports/global-oncology-trends-2018](about:blank). Accessed January 20, 2020.
14. OECD. *New Health Technologies: Managing Access, Value and Sustainbility.* Paris: OECD Publishing; 2017.
15. The World Bank. New country classifications by income level: 2019-2020. 2019; [https://blogs.worldbank.org/opendata/new-country-classifications-income-level-2019-2020](about:blank). Accessed January 02, 2020.
16. Bradley EH, Curry LA, Devers KJ. Qualitative data analysis for health services research: developing taxonomy, themes, and theory. *Health Serv Res.* 2007;42(4):1758-1772.
17. European Commission. HTA network reflection paper on “synergies between regulatory and hta issues on pharmaceuticals. 2016; [https://ec.europa.eu/health/sites/health/files/technology_assessment/docs/ev_20161110_co06_en.pdf](about:blank). Accessed March 30, 2020, European Commission.
18. Enzmann H. New trends and challenges in the European regulation of innovative medicines. *Regul Toxicol Pharmacol.* 2016;80:314-320.
19. Liberti L, Pichler F, Walker S. Preparing for regulator review and reimbursement decisions- a case for cooperation between regulatory authorities, sponsors and health technology assessment agencies. . *Pharm Med* 2009;23:263-267.
20. Henshall C, Mardhani-Bayne L, Fronsdal KB, Klemp M. Interactions between health technology assessment, coverage, and regulatory processes: emerging issues, goals, and opportunities. *Int J Technol Assess Health Care.* 2011;27(3):253-260.
21. Office of Health Economics (OHE). Workshop on HTA and Regulatory Review. 2010; [https://www.ohe.org/news/workshop-hta-and-regulatory-review](about:blank). Accessed January 15 2020.
22. Hutton J, Trueman P, Facey K. Harmonization of evidence requirements for health technology assessment in reimbursement decision making. *Int J Technol Assess Health Care.* 2008;24(4):511-517.
23. European Medicines Agency. Parallel consultation with regulators and health technology assessment bodies. 2019; [https://www.ema.europa.eu/en/human-regulatory/research-development/scientific-advice-protocol-assistance/parallel-consultation-regulators-health-technology-assessment-bodies](about:blank). Accessed January 15 2020.
24. Balaisyte L, Joos A, Hiligsmann M. Early Dialogue in Europe: Perspectives on Value, Challenges, and Continuing Evolution. *Int J Technol Assess Health Care.* 2018;34(5):514-518.
25. Wang T, McAuslane N, Liberti L, Leufkens H, Hovels A. Building Synergy between Regulatory and HTA Agencies beyond Processes and Procedures-Can We Effectively Align the Evidentiary Requirements? A Survey of Stakeholder Perceptions. *Value Health.* 2018;21(6):707-714.
26. Messner DA, Towse A, Mohr P, Garau M. The future of comparative effectiveness and relative efficacy of drugs: an international perspective. *J Comp Eff Res.* 2015;4(4):419-427.
27. Institute of Medicine Committee on Comparative Effectiveness Research Prioritization. *Initial National Priorities for Comparative Effectiveness Research.* Washington, DC, USA: The National Academies Press; 2009.
28. Jonsson B. Relative effectiveness and the European pharmaceutical market. *Eur J Health Econ.* 2011;12(2):97-102.
29. Eichler HG, Abadie E, Breckenridge A, et al. Bridging the efficacy-effectiveness gap: a regulator's perspective on addressing variability of drug response. *Nat Rev Drug Discov.* 2011;10(7):495-506.
30. Wildson T, A S. *A comparative analysis of the role and impact of Health Technology Assessment* Charles River Associates; 2011.
31. Towse A, Jonsson B, McGrath C, et al. UNDERSTANDING VARIATIONS IN RELATIVE EFFECTIVENESS: A HEALTH PRODUCTION APPROACH. *International journal of technology assessment in health care.* 2015;31(6):363-370.
32. Connolly SJ, Ezekowitz MD, Yusuf S, et al. Dabigatran versus warfarin in patients with atrial fibrillation. *N Engl J Med.* 2009;361(12):1139-1151.
33. Garber AM, Sox HC. The role of costs in comparative effectiveness research. *Health Aff (Millwood).* 2010;29(10):1805-1811.
34. Myshko D. Addressing Reimbursement During Development. 2012; [https://www.pharmavoice.com/article/2012-05-addressing-reimbursement-during-development/](about:blank). Accessed January 12 2020.
35. Miller HI. A proposal for FDA reform. *Nat Rev Drug Discov.* 2002;1(8):642-648.
36. Woodcock J. Comparative effectiveness research and the regulation of drugs, biologics and devices. *J Comp Eff Res.* 2013;2(2):95-97.
37. Patient Protection and Affordable Care Act. Public Law 111–148. [www.gpo.gov/fdsys/pkg/BILLS-111hr3590enr/pdf/BILLS-111hr3590enr.pdf](about:blank). Accessed January 08, 2020.
38. Messner DA, Mohr P, Towse A. Futurescapes: evidence expectations in the USA for comparative effectiveness research for drugs in 2020. *J Comp Eff Res.* 2015;4(4):385-400.
39. Blackstone EA, Fuhr JP, Jr., Ziernicki D. Will comparative effectiveness research finally succeed? *Biotechnol Healthc.* 2012;9(3):22-26.
40. CTMP. Green Park Collaborative. 2020; [http://www.cmtpnet.org/green-park-collaborative/](about:blank). Accessed January 12, 2020.
41. European Commission. High level pharmaceutical forum 2005-2008 conclusions and recommendations. 2008; [https://op.europa.eu/en/publication-detail/-/publication/4fddf639-47cc-4f90-9964-142757d2515a](about:blank). Accessed Februray 03, 2020.
42. Berntgen M, Gourvil A, Pavlovic M, Goettsch W, Eichler HG, Kristensen FB. Improving the contribution of regulatory assessment reports to health technology assessments--a collaboration between the European Medicines Agency and the European network for Health Technology Assessment. *Value Health.* 2014;17(5):634-641.
43. European Medicines Agency. EMA-EUnetHTA three-year work plan. 2013; [https://www.ema.europa.eu/en/documents/other/european-medicines-agency-eunethta-three-year-work-plan_en.pdf](about:blank). Accessed January 09, 2020.
44. European Medicines Agency. Report on the implementation of the EMA-EUnetHTA threeyear work plan 2012-2015. 2016; [https://www.ema.europa.eu/en/documents/report/report-implementation-european-medicines-agency-european-network-health-technology-assessment_en.pdf](about:blank). Accessed January 23, 2020.
45. Tafuri G, Pagnini M, Moseley J, et al. How aligned are the perspectives of EU regulators and HTA bodies? A comparative analysis of regulatory-HTA parallel scientific advice. *Br J Clin Pharmacol.* 2016;82(4):965-973.
46. Dekker M, Bouvy JC, O'Rourke D, et al. Alignment of European Regulatory and Health Technology Assessments: A Review of Licensed Products for Alzheimer's Disease. *Front Med (Lausanne).* 2019;6:73.
47. U.S. Department of Health and Human Services Food and Drug Administration. Best Practices for Communication Between IND Sponsors and FDA During Drug Development Guidance for Industry and Review Staff Good Review Practice 2017; [https://www.fda.gov/media/94850/download](about:blank). Accessed March 25, 2019.
48. European Medicines Agency. *European Medicines Agency guidance for applicants seeking scientific advice and protocol assistance* London: EMA; 2017.
49. Hofer MP, Jakobsson C, Zafiropoulos N, et al. Regulatory watch: Impact of scientific advice from the European Medicines Agency. *Nat Rev Drug Discov.* 2015;14(5):302-303.
50. Backhouse ME, Wonder M, Hornby E, Kilburg A, Drummond M, Mayer FK. Early dialogue between the developers of new technologies and pricing and reimbursement agencies: a pilot study. *Value Health.* 2011;14(4):608-615.
51. Fronsdal K, Pichler F, Mardhani-Bayne L, et al. Interaction initiatives between regulatory, health technology assessment and coverage bodies, and industry. *Int J Technol Assess Health Care.* 2012;28(4):374-381.
52. European Medicines Agency. *EMA-HTA workshop Bringing together stakeholders for early dialogue in medicines development:Report from the public workshop hosted by the European Medicines Agency (EMA) in London on 26 November 2013.* London: EMA; 2013.
53. Khan S, Carter M. European Medicines Agency-Health Technology Assessment Parallel Consultation Platform: An Industry Perspective. *Clin Pharmacol Ther.* 2019;105(4):822-825.
54. European Medicines Agency. Best Practice guidance for Pilot EMA HTA Parallel Scientific Advice procedures. 2014; [https://www.ema.europa.eu/en/documents/regulatory-procedural-guideline/draft-best-practice-guidance-pilot-european-medicines-agency-health-technology-assessment-parallel_en.pdf](about:blank). Accessed January 15, 2020.
55. Tschank D. *The Impact of the EMA-EUnetHTA Collaboration on Drug Development*: der Mathematisch-Naturwissenschaftlichen Fakultät, Friedrich-Wilhelms-Universität Bonn; 2014.
56. Bramley T, Spackman E, Hebborn A, Ho LK, Parcher B, McLaughlin T. To Seek or Not to Seek Parallel European Medicine Agency and Health Technology Assessment Scientific Advice? *Value & Outcomes Spotlight* 2017(March/April):12-15.
57. Tafuri G, Lucas I, Estevao S, et al. The impact of parallel regulatory-health technology assessment scientific advice on clinical development. Assessing the uptake of regulatory and health technology assessment recommendations. *Br J Clin Pharmacol.* 2018;84(5):1013-1019.
58. NICE. Scientific Advice. 2020; [https://www.nice.org.uk/about/what-we-do/life-sciences/scientific-advice](about:blank). Accessed January 03, 2020.
59. Trapestry Network. Pilots of multi-country, multi-stakeholder consultations in drug development: from proof of concept to tangible benefits. 2012; [https://www.tapestrynetworks.com/sites/default/files/publication_pdf/Pilots-of-multi-stakeholder-consultations-in-drug-development-6-June-2012.pdf](about:blank). Accessed February 05, 2020.
60. Wonder M, Backhouse ME, Hornby E. Early scientific advice obtained simultaneously from regulators and payers: findings from a pilot study in Australia. *Value Health.* 2013;16(6):1067-1073.
61. McAuslane N, Liberti L, Connelly P. The Confluence of Accelerated Regulatory and Health Technology Assessment Access Pathways. *Clin Pharmacol Ther.* 2019;105(4):935-942.
62. Australian Government. Memorandum of Understanding 2010; [http://www.pbs.gov.au/industry/useful-resources/memorandum/MOU-MA-Sep2010.pdf](about:blank). Accessed January 12, 2020.
63. Department of Health. TGA and PBAC Parallel Process and Requirements. 2018; [http://www.pbs.gov.au/info/publication/factsheets/shared/tga-pbac-parallel-process](about:blank). Accessed March 25, 2020.
64. Pearce A, van Gool K, Haywood P, Haas M. Delays in access to affordable medicines: putting policy into perspective. *Aust Health Rev.* 2012;36(4):412-418.
65. Centre for Innovation in Regulatory Science (CIRS). *Review of HTA outcomes and timelines in Australia, Canada and Europe 2014-2018.* London: CRIS; 2019.
66. GlaxoSmithKline Australia Pty Ltd, ViiV Healthcare Pty Ltd. The Pharmaceutical Benefi ts Scheme in Australia: An explainer on system components. 2018; [https://au.gsk.com/media/421635/gsk-viiv-the-pbs-in-australia-feb-2018.pdf](about:blank). Accessed January 21, 2020.
67. Centre for Innovation in Regulatory Science (CIRS). *Review of HTA outcomes and timelines in Australia, Canada and Europe 2014-2015.* London: CRIS; 2017.
68. CADTH. CADTH Common Drug Review Will Accept Submissions Up to Six Months Pre-Notice of Compliance (NOC). 2018; [https://cadth.ca/news/cadth-common-drug-review-will-accept-submissions-six-months-pre-notice-compliance-noc](about:blank). Accessed January 15, 2020.
69. Liberti L. Will parallel regulatory and HTA review processes reduce access time for patients in Canada? 2018; [https://www.cadth.ca/sites/default/files/symp-2018/presentations/april16-2018/Concurrent-Session-B1-Lawrence-Liberti.pdf](about:blank). Accessed January 12, 2020.
70. Federal Register. Parallel Review of Medical Products. 2010; [https://www.federalregister.gov/documents/2010/09/17/2010-23252/parallel-review-of-medical-products](about:blank). Accessed February 12, 2020.
71. Podemska-Mikluch M. *FDA-CMS Parallel Review: A Failed Attempt at Spurring Innovation.* Arlington, VA: Mercatus Center at George Mason University, ; 2016.
72. Ridge JR, Statz S. Exact Sciences' experience with the FDA and CMS parallel review program. *Expert Rev Mol Diagn.* 2015;15(9):1117-1124.
73. Bhatt DL, Kandzari DE, O'Neill WW, et al. A controlled trial of renal denervation for resistant hypertension. *N Engl J Med.* 2014;370(15):1393-1401.
74. Federal Register. Program for Parallel Review of Medical Devices. 2016; [https://www.federalregister.gov/documents/2016/10/24/2016-25659/program-for-parallel-review-of-medical-devices](about:blank). Accessed January 11, 2020.
75. Mezher M. FDA, CMS: Second Parallel Review Decision Ever for NGS Test. 2017; [https://www.raps.org/regulatory-focus%E2%84%A2/news-articles/2017/12/fda,-cms-second-parallel-review-decision-ever-for-ngs-test](about:blank). Accessed January 23, 2020.
76. Netherlands Healthcare Institute Pilot 'Parallel Procedures MEB-ZIN'. 2020; [https://www.zorginstituutnederland.nl/werkagenda/parallelle-procedures-cbg-zin](about:blank). Accessed April 25, 2020.
77. MaRS Discovery District. Why the medical device industry is shifting focus to early health technology assessment. 2020; [https://www.marsdd.com/news/why-the-medical-device-industry-is-shifting-focus-to-early-health-technology-assessment/](about:blank). Accessed January 15, 2020.
78. Bouvy JC, Jonsson P, Longson C, Crabb N, Garner S. Health Technology Assessment in the Context of Adaptive Pathways for Medicines in Europe: Challenges and Opportunities. *Clin Pharmacol Ther.* 2016;100(6):594-597.
79. Brownsword R, Scotford E, Yeung K. *The Oxford Handbook of Law, Regulation, and Technology.* Oxford, UK: Oxford University Press; 2017.
80. Eichler HG, Oye K, Baird LG, et al. Adaptive licensing: taking the next step in the evolution of drug approval. *Clin Pharmacol Ther.* 2012;91(3):426-437.
81. Nicotera G, Sferrazza G, Serafino A, Pierimarchi P. The Iterative Development of Medicines Through the European Medicine Agency's Adaptive Pathway Approach. *Front Med (Lausanne).* 2019;6:148.
82. Eichler HG, Baird LG, Barker R, et al. From adaptive licensing to adaptive pathways: delivering a flexible life-span approach to bring new drugs to patients. *Clinical pharmacology and therapeutics.* 2015;97(3):234-246.
83. Stein D, Soni M. Early Access Programs Opportunities and Challenges for Real-World Data Collection. 2018; [https://www.evidera.com/wp-content/uploads/2018/05/Early-Access-Programs-Real-World-Data.pdf](about:blank). Accessed January 12, 2020.
84. European Medicines Agency. PRIME: priority medicines 2020; [https://www.ema.europa.eu/en/human-regulatory/research-development/prime-priority-medicines](about:blank). Accessed March 22, 2020.
85. European Medicines Agency. Conditional marketing authorisation 2020; [https://www.ema.europa.eu/en/human-regulatory/marketing-authorisation/conditional-marketing-authorisation](about:blank). Accessed April 02, 2020.
86. European Medicines Agency. Compassionate use 2020; [https://www.ema.europa.eu/en/human-regulatory/research-development/compassionate-use](about:blank). Accessed January 12, 2020.
87. European Medicines Agency. Final report on the adaptive pathways pilot. 2020; [https://www.ema.europa.eu/en/documents/report/final-report-adaptive-pathways-pilot_en.pdf](about:blank). Accessed February 16, 2020.
88. National Institute for Public Health and the Environment. *The adaptive pathways process: benefits and potential hurdles from a Dutch perspective.* Bilthoven, The Netherlands: RIVM; 2016.
89. Baird LG, Trusheim MR, Eichler HG, Berndt ER, Hirsch G. Comparison of Stakeholder Metrics for Traditional and Adaptive Development and Licensing Approaches to Drug Development. *Ther Innov Regul Sci.* 2013;47(4):474-483.
90. Innovative Medicines Initiative. ADAPTSMART. 2020; [http://adaptsmart.eu/adapt-smart-kick-off-meeting-the-innovative-medicines-initiatives-adapt-smart-laying-the-foundations-and-building-consensus-to-make-mapps-work-for-all/](about:blank). Accessed March 15, 2020.
91. Oye K, Baird LG, Chia A, et al. Legal foundations of adaptive licensing. *Clin Pharmacol Ther.* 2013;94(3):309-311.
92. Schulthess D, Baird LG, Trusheim M, et al. Medicines Adaptive Pathways to Patients (MAPPs): A Story of International Collaboration Leading to Implementation. *Ther Innov Regul Sci.* 2016;50(3):347-354.
93. European Medicines Agency. European Medicines Agency post-authorisation procedural advice for users of the centralised procedure 2019; [https://www.ema.europa.eu/en/documents/regulatory-procedural-guideline/european-medicines-agency-post-authorisation-procedural-advice-users-centralised-procedure_en.pdf](about:blank). Accessed March 12, 2020.
94. Food and Drug Administration. Postmarketing Studies and Clinical Trials - Implementation of section 505(o)(3) of the Federal Court Food, Drug and Cosmetic Act. 2011. 2011; [http://www.fda.gov/downloads/Drugs/GuidanceComplianceRegulatoryInformation/Guidances/UCM172001.pdf](about:blank). Accessed March 13, 2020.
95. European Network of Centres for Pharmacoepidemiology and Pharmacovigilance. ENCePP Guide on Methodological Standards in Pharmacoepidemiology. 2018; [http://www.encepp.eu/standards_and_guidances/methodologicalGuide.shtml](about:blank). Accessed January 12, 2020.
96. Tavridou A, Sarinic V. PRAC interaction with SAWP PASS pilot and other consultations. 2016; [https://www.ema.europa.eu/en/documents/presentation/presentation-prac-interaction-sawp-anna-tavridou_en.pdf](about:blank). Accessed April 25, 2020.
97. McGettigan P, Olmo CA, et al. Patient Registries: An Underused Resource for Medicines Evaluation: Operational proposals for increasing the use of patient registries in regulatory assessments. Drug Safety. 2019 Nov;42(11):1343-1351.
98. European Medicines Agency. Patient Registries. 2018; [https://www.ema.europa.eu/en/human-regulatory/post-authorisation/patient-registries](about:blank). Accessed January 15, 2020.
99. Get Real. Overall objectives. 2020; [https://www.imi-getreal.eu/About-GetReal/Overall-objectives](about:blank). Accessed January 16, 2020.
100. Tsoi B, O'Reilly D, Masucci L, Drummond M, Goeree R. Harmonization of HTA--based reimbursement and regulatory approval activities: a qualitative study. *J Popul Ther Clin Pharmacol.* 2015;22(1):e78-89.
101. CADTH. Procedures for the CADTH Common Drug Review and Interim Plasma Protein Product Review. 2020; [https://cadth.ca/sites/default/files/cdr/process/Procedure_and_Guidelines_for_CADTH_CDR.pdf](about:blank). Accessed March 25, 2020.
102. Swedish Medical Products Agency. Scientific Advice. 2019; [https://www.lakemedelsverket.se/en/permission-approval-and-control/advisory/scientific-advice](about:blank). Accessed January, 15, 2020.
